# Novel Fusarium wilt resistance genes uncovered in natural and cultivated strawberry populations are found on three non-homoeologous chromosomes

**DOI:** 10.1007/s00122-022-04102-2

**Published:** 2022-05-18

**Authors:** Dominique D. A. Pincot, Mitchell J. Feldmann, Michael A. Hardigan, Mishi V. Vachev, Peter M. Henry, Thomas R. Gordon, Marta Bjornson, Alan Rodriguez, Nicolas Cobo, Randi A. Famula, Glenn S. Cole, Gitta L. Coaker, Steven J. Knapp

**Affiliations:** 1 Department of Plant Sciences, One Shields Avenue, University of California, Davis, CA 95616 USA; 2 Horticultural Crops Research Unit, United States Department of Agriculture, Agricultural Research Service, Corvallis, OR 97331 USA; 3 United States Department of Agriculture Agricultural Research Service, 1636 East Alisal Street, Salinas, CA 93905 USA; 4 Department of Plant Pathology, One Shields Avenue, University of California, Davis, CA 95616 USA; 5 Departamento de Producción, Agropecuaria Universidad de La Frontera, Temuco, Chile

## Abstract

**Key Message:**

**Several Fusarium wilt resistance genes were discovered, genetically and physically mapped, and rapidly deployed via marker-assisted selection to develop cultivars resistant to** ***Fusarium oxysporum*** **f. sp.** ***fragariae***, **a devastating soil-borne pathogen of strawberry**.

**Abstract:**

Fusarium wilt, a soilborne disease caused by *Fusarium oxysporum* f. sp. *fragariae*, poses a significant threat to strawberry (*Fragaria* $\times$ *ananassa*) production in many parts of the world. This pathogen causes wilting, collapse, and death in susceptible genotypes. We previously identified a dominant gene (*FW1*) on chromosome 2B that confers resistance to race 1 of the pathogen, and hypothesized that gene-for-gene resistance to Fusarium wilt was widespread in strawberry. To explore this, a genetically diverse collection of heirloom and modern cultivars and octoploid ecotypes were screened for resistance to Fusarium wilt races 1 and 2. Here, we show that resistance to both races is widespread in natural and domesticated populations and that resistance to race 1 is conferred by partially to completely dominant alleles among loci (*FW1*, *FW2*, *FW3*, *FW4*, and *FW5*) found on three non-homoeologous chromosomes (1A, 2B, and 6B). The underlying genes have not yet been cloned and functionally characterized; however, plausible candidates were identified that encode pattern recognition receptors or other proteins known to confer gene-for-gene resistance in plants. High-throughput genotyping assays for SNPs in linkage disequilibrium with *FW1*-*FW5* were developed to facilitate marker-assisted selection and accelerate the development of race 1 resistant cultivars. This study laid the foundation for identifying the genes encoded by *FW1-FW5*, in addition to exploring the genetics of resistance to race 2 and other races of the pathogen, as a precaution to averting a Fusarium wilt pandemic.

## Introduction

*Fusarium oxysporum*, a widespread soil-borne pathogen, causes vascular wilt disease in several economically important plants (Michielse and Rep 2009; Dean et al. 2012), in addition to the broad spectrum human disease known as ‘fusariosis’ (Dignani and Anaissie 2004; Nucci and Anaissie 2007; Batista et al. 2020). *F. oxysporum* is one of the most destructive plant-pathogenic fungi worldwide, with a long and storied history of outbreaks and epidemics that have caused significant production losses and disrupted food and fiber production (Dean et al. 2012). One of the earliest reports of the disease arose from outbreaks on banana (*Musa acuminata* Colla) in the late 1800s that progressively annihilated the widely grown susceptible cultivar ‘Gros Michel’, forced the abandonment of export plantations, and caused a gradual, albeit inexorable shift in production from susceptible ‘Gros Michel’ to resistant ‘Cavendish’ cultivars (Ploetz 2015; Dale et al. 2017; Pegg et al. 2019). Similar production shifts have unfolded over the last century in tomato (*Solanum lycopersicum* L.), cotton (*Gossypium hirsutum* L.), and other economically important plants (Michielse and Rep 2009), and more recently strawberry (*Fragaria* $\times$ *ananassa* Duchesne ex Rozier) (Pincot et al. 2018; Henry et al. 2021). The discovery of sources of resistance and development and deployment of resistant cultivars has been critical for limiting disease losses and sustaining agricultural production in strawberry and other host plants affected by the pathogen (Dean et al. 2012; Gordon 2017; Pincot et al. 2018; Henry et al. 2019).

Fusarium wilt of strawberry is caused by *F. oxysporum* f. sp. *fragariae* (Fof), one of more than 100 documented host-specific pathogens (formae speciales), many of which have been widely disseminated (Gordon 2017). Although the strawberry-specific Fof has been reported in many countries, the disease has been most widely reported and studied in Japan, South Korea, Australia, and California, between which virulent strains have been disseminated (Gordon 2017; Henry et al. 2017, 2021). Fusarium wilt was first reported on strawberry in Australia in the 1960s (Winks and Williams 1965), and was not reported on strawberry in California until the mid-2000s (Koike et al. 2009; Koike and Gordon 2015). The disease has been aggressively spreading and poses a serious threat to production in California (Koike and Gordon 2015; Henry et al. 2017, 2019).

Fusarium wilt has not yet become a serious threat to production everywhere strawberries are grown; however, there is a significant risk of virulent strains being disseminated through global trade, and the ever present danger of the evolution and emergence of virulent races of the pathogen that defeat known resistance (*R*) genes (Henry et al. 2021). One of the motivations for the present study was to prepare for that inevitability by delving more deeply into the genetics of resistance and developing the resources and knowledge needed to accelerate the development of Fusarium wilt resistant cultivars through marker-assisted selection (MAS). To that end, we initiated studies in 2015 to identify sources of resistance to California isolates of the pathogen and shed light on the genetics of resistance to Fusarium wilt in strawberry (Pincot et al. 2018). The prevalence, diversity, strength of resistance, and genetic mechanisms underlying resistance to Fusarium wilt were unknown when those studies were initiated (Mori et al. 2005; Paynter et al. 2014; Pincot et al. 2018). Significant insights into the *Fragaria-Fusarium* pathosystem have since emerged.

Pincot et al. (2018) identified multiple sources of resistance to Fusarium wilt in a closed breeding population developed at the University of California, Davis (hereafter designated as the ’California’ population). The isolate they used (AMP132) was subsequently classified as Fof race 1 (Henry et al. 2021). From the resistance phenotypes of plants artificially inoculated with AMP132, they observed a nearly bimodal distribution of resistant and susceptible individuals in a genome-wide association study (GWAS) of the California population, observed near-Mendelian distributions for resistance phenotypes in segregating populations, and showed that resistance to AMP132 was conferred by a single dominant gene (*FW1*) in the California population. The resistant allele (*FW1*) had a low frequency (0.16) and was only homozygous in 3% of the resistant individuals in the California population (Pincot et al. 2018). From analyses of pedigree records and haplotypes of SNP markers in linkage disequilibrium with the *FW1* locus, Pincot et al. (2018) predicted that 99% of the resistant individuals in the California population carried *FW1*. They concluded that the resistant allele (*FW1*) had fortuitously survived early breeding bottlenecks and originated in the earliest known ancestors of the California population (Pincot et al. 2018, 2021; Hardigan et al. 2021a).

Pincot et al. (2018) screened two non-California cultivars (Guardian and Earliglow), both of which were shown to be resistant to race 1 and had SNP marker haplotypes different from the *FW1* SNP marker haplotype. The only AMP132-resistant cultivar in the California population without the *FW1* SNP marker haplotype was the heirloom cultivar ‘Wiltguard’. We speculated that Earliglow, Guardian, and Wiltguard might carry novel *R*-genes, a hypothesis tested in the present study. To build on earlier findings in the California population and develop a deeper understanding of the genetics of resistance, we screened a diverse collection of elite and exotic germplasm accessions (clonally preserved individuals) for resistance to race 1 and selected several additional race 1 resistant donors for further study. Here, we show that resistance to race 1 is widespread in elite and exotic germplasm, including geographically diverse ecotypes of the wild octoploid progenitors of strawberry (*F. chiloensis* and *F. virginiana*).

Plant genes that confer strong race-specific resistance frequently encode proteins with nucleotide-binding leucine-rich repeat domains (NBS-LRRs or NLRs) or surface localized pattern recognition receptors (PRRs) (Lolle et al. 2020). Several of the previously described Fusarium wilt *R*-genes encode proteins with NLR and PRR architecture (Ori et al. 1997; Joobeur et al. 2004; Diener and Ausubel 2005; Michielse and Rep 2009; Lv et al. 2014; Catanzariti et al. 2015; Gonzalez-Cendales et al. 2016; Catanzariti et al. 2017). *R*-genes that confer resistance to *F. oxysporum* f. sp. *lycopersici* in tomato (*I*, *I-2*, *I-3*, *I-4*, and *I-7*) are among the most well studied examples (Sela-Buurlage et al. 2001; Hemming et al. 2004; Houterman et al. 2009; Michielse and Rep 2009). PRRs are capable of recognizing conserved pathogen features and extracellular effectors, while NLR receptors recognize secreted pathogen effectors inside plant cells, resulting in disease resistance (Jones et al. 2016; Albert et al. 2020; Lolle et al. 2020). Although the gene encoded by *FW1* has not yet been identified, we posited that *FW1* might encode an NLR or PRR immune receptor protein that recognizes an effector protein encoded by Fof race 1 isolates (called *AvrFW1*).

Because *R*-genes often have short-lived utility (Mundt 2014), the continual discovery and deployment of novel *R*-genes has been critical for keeping pace with the evolution of pathogen races in the gene-for-gene ‘arms race’ (Hammond-Kosack and Jones 1996, 1997; Boller and He 2009; Dangl et al. 2013; Chiang and Coaker 2015). The durability of *FW1* and other race-specific *R*-genes is uncertain (Mundt 2014, 2018), and depends on the speed of emergence of novel Fof races through pathogen mutation (White et al. 2000; Rouxel and Balesdent 2010; Henry et al. 2021). If *FW1* encodes an NLR or PRR, a mutation of *AvrFW1* could lead to an evasion of host immune perception and regained pathogenicity (Zhang and Coaker 2017). Currently, only race 1 isolates of Fof have been found in California, and none cause disease in cultivars carrying the dominant *FW1* allele (Henry et al. 2017, 2021; Pincot et al. 2018). However, race 2 isolates that cause disease on *FW1*-carrying cultivars have been observed (Henry et al. 2021). The identification of race 2 reinforces the expectation that novel strains of the pathogen could eventually evolve and defeat defeat race 1 *R*-genes through mutation, loss, or expression polymorphism in *AvrFW1*.

The identification of *FW1* and *AvrFW1* and advances in the development of genomic resources for *Fragaria* and *Fusarium* laid the foundation for the present study. *FW1* was originally discovered by GWAS using a diploid reference genome (Pincot et al. 2018). The approximate location of *FW1* in the octoploid genome was subsequently ascertained by genetic mapping in octoploid segregating populations genotyped with a single nucleotide polymorphsim (SNP) array designed with probe DNA sequences anchored to a diploid reference genome (Bassil et al. 2015; Verma et al. 2017; Pincot et al. 2018). The octoploid genome has since been sequenced (Edger et al. 2019; Hardigan et al. 2021b), thereby opening the way for octoploid genome-informed breeding and genetic studies in strawberry. Those genome assemblies supplied the foundation for several additional technical advances, the most important of which were the genome-wide discovery and physical and genetic mapping of millions of DNA variants in the octoploid genome, the development of 50K and 850K SNP genotyping arrays with probe DNA sequences uniformly distributed and anchored to physical positions throughout the octoploid genome, and telomere-to-telomere resolution of the A, B, C, and D subgenomes of octoploid strawberry (Hardigan et al. 2020, 2021a). These breakthroughs and resources were critical for the present study, which included: (a) pinpointing the genomic location of the *FW1* locus and four newly discovered Fusarium wilt resistance loci (*FW2*, *FW3*, *FW4*, and *FW5*); (b) expanding the database of octoploid germplasm accessions screened for resistance to Fusarium wilt races 1 and 2; (c) identifying SNPs and other DNA variants in linkage disequilibrium with *FW1*-*FW5*; and (d) identifying plausible candidate genes for *FW1*-*FW5* through genotype-to-phenotype associations. Finally, we describe high-throughput genotyping assays for SNPs in strong LD with *FW1*-*FW5* to facilitate the development of Fusarium wilt resistant cultivars through MAS.

## Materials and methods

### Plant material

The plant materials for our studies included 309 *F.* $\times$ *ananassa*, 62 *F. chiloensis*, and 40 *F. virginiana* germplasm accessions (individuals) preserved in the University of California, Davis (UC Davis) Strawberry Germplasm Collection or the United States Department of Agriculture, Agricultural Research Service, National Plant Germplasm System (USDA-ARS NPGS), National Clonal Germplasm Repository, Corvallis, Oregon (https://www.ars-grin.gov/). The original ‘mother’ plants of individuals acquired from the USDA were asexually multiplied in a Winters, CA field nursery and preserved in the UC Davis Strawberry Germplasm Collection throughout the course of our studies (Online Resource 1). Bare-root plants (clones) of every individual were produced by asexual multiplication in high-elevation (1294 m) field nurseries in Dorris, CA from mother plants propagated in low-elevation (41 m) field nurseries in Winters, CA. The mother plants were planted mid-April and daughter plants were harvested and trimmed in mid-October and stored in plastic bags at 3.5 °C for two to three weeks before pathogen inoculation and planting. The daughter plants for growth chamber and greenhouse experiments were stored at –2.2 °C for 5 to 27 weeks and ultimately thawed and stored at 3.5 °C for one to three days prior to pathogen inoculation and planting.

$\hbox {S}_1$ families were developed by self-pollinating three Fusarium wilt race 1 resistant *F.* $\times$ *ananassa* cultivars identified by Pincot et al. (2018): Guardian (PI551407), Wiltguard (PI551669; 52C016P007), and Earliglow (PI551394). An $\hbox {S}_1$ family was developed by self-pollinating a resistant individual (17C327P010) we identified in a population developed by crossing the susceptible cultivar Cabrillo with the resistant *F. virginiana* subsp. *glauca* ecotype PI612500. An $\hbox {S}_2$ family was developed by self-pollinating 61S016P006, a highly resistant $\hbox {S}_1$ individual identified in our resistance screening study. These individuals were known from genome-wide DNA profiling to be highly heterozygous and predicted *a priori* to either be heterozygous or homozygous for alleles affecting resistance. We developed interspecific full-sib families by crossing a susceptible *F.* $\times$ *ananassa* parent (12C089P002) with race 1 resistant ecotypes of *Fragaria virginiana* subsp. *virginiana* (PI552277), *Fragaria chiloensis* subsp. *patagonica* (PI602575), and *Fragaria virginiana* subsp. *grayana* (PI612569). These ecotypes were identified in the present study, known to be highly heterozygous from genome-wide DNA profiling, and, as before, predicted *a priori* to either be heterozygous or homozygous for alleles affecting resistance. The parents of these populations were grown in greenhouses at UC Davis. $\hbox {S}_1$ and $\hbox {S}_2$ family seeds were produced by hand pollinating unemasculated flowers of Guardian, Wiltguard, Earliglow, 17C327P010, and 61S016P006. The PI612569 $\times$ 12C089P002, 12C089P002 $\times$ PI602575, and PI552277 $\times$ 12C089P002 full-sib families were produced by emasculating flowers on greenhouse grown plants of the female parent and hand pollinating the emasculated flowers with pollen from male parents. Ripe fruit were harvested and macerated in a pectinase solution (0.6 g/L) to separate achenes (seeds) from receptacles. Seeds were scarified by soaking in a 36 normal sulfuric acid solution for 16 min. Scarified seeds were germinated on moistened blotter paper at room temperature (approximately 22–24 °C). Seedlings were transplanted to sterilized soil and were greenhouse grown for 9 months in Winters, CA before transplanting to the field, or were grown in a growth chamber for two to four months in Davis, CA before transplanting to the greenhouse.

### Artificial inoculation protocols and disease resistance phenotyping

The plants for our experiments were artificially inoculated with race 1 (AMP132) or 2 (MAFF727510) isolates of *F. oxysporum* f. sp. *fragariae* using previously described protocols (Pincot et al. 2018; Henry et al. 2021). The AMP132 isolate originated in California, whereas the MAFF727510 isolate originated in Japan (Gordon et al. 2016; Henry et al. 2017, 2021; Pincot et al. 2018). To produce spores, the pathogen was grown on potato dextrose agar or Kerr’s broth under continuous fluorescent lighting at room temperature, as previously described (Pincot et al. 2018; Henry et al. 2021). Crude suspensions were passed through two layers of sterilized cheesecloth to remove hyphae. Spore densities were estimated using a haemocytometer and diluted with either sterile DI water (AMP132) or 0.1% water agar (MAFF727510) to a final density of 5 $\times$ 10^6^ spores/ml. Seedling and bare-root plants were inoculated by submerging their root systems up to the crown in the spore suspension for 7–8 min prior to planting.

The individuals in these studies were visually phenotyped for resistance to Fusarium wilt over multiple post-inoculation time points using an ordinal disease rating scale from 1 (highly resistant) to 5 (highly susceptible) (Gordon et al. 2016; Henry et al. 2017, 2021; Pincot et al. 2018). For our field studies, individual plants were phenotyped once per week for four to eight consecutive weeks beginning in early June. Symptoms were observed on plants 26- to 36-weeks post-inoculation. For greenhouse and growth chamber studies, entries were phenotyped weekly for 6 to 12 weeks post-inoculation. For field, greenhouse, and growth chamber experiments, the onset and progression of disease symptoms among resistant and susceptible checks were used as guides for initiating and terminating phenotyping.

### Race 1 resistance screening experiments

Our race 1 resistance screening experiments were conducted over a three year period (2016–17 to 2018–19) at the UC Davis Plant Pathology Farm. The plants for these experiments were artificially inoculated with the AMP132 isolate of the pathogen. Strawberries had not been previously grown in the fields selected for our studies. The fields were tilled and disked prior to fumigation and were broadcast-fumigated in October of each year with a 60:40 mixture of chloropicrin:1,3-dichloropropene (Pic-Clor 60, Cardinal Professional Products, Woodland, CA) at 560.4 kg/ha. The entire field was sealed with an impermeable plastic film for one-week post-fumigation before shaping 15.3 cm tall $\times$ 76.2 cm center-to-center raised beds. Sub-surface irrigation drip tape was installed longitudinally along the beds followed by black plastic mulch with a single row of planting holes spaced 30.5 cm apart. Artificially inoculated plants were transplanted in mid-November both years. The fields were fertilized with approximately 198 kg/ha of nitrogen over the growing season and irrigated as needed to prevent water stress.

For the 2016–17 field experiment, 344 germplasm accessions (identified in Online Resource 1) were screened for resistance to AMP132 and were part of a study that included 565 germplasm accessions developed at UC Davis, which is hereafter identified as the ‘California’ population. The resistance phenotypes for the latter were previously reported by Pincot et al. (2018). Collectively, 981 germplasm accessions were screened in the 2016-17 field study. These were arranged in a square lattice experiment design with four single-plant replicates per entry (Hinkelmann and Kempthorne 1994). The experiment design and randomizations of entries within incomplete blocks were generated with the R package *agricolae* (De Mendiburu 2015). For the 2017-18 and 2018-19 field experiments, 144 ‘host differential panel’ individuals were screened for resistance to AMP132 (identified in Online Resource 1). These individuals were arranged in a 12 $\times$ 12 square lattice experiment design with four single-plant replicates per entry as described above.

Guardian, Wiltguard, and Earliglow $\hbox {S}_1$ and 61S016P006 $\hbox {S}_2$ populations were screened for resistance to AMP132 in the 2016-17 field study. Ninety-nine Guardian $\hbox {S}_1$ and 98 Wiltguard $\hbox {S}_1$ individuals were phenotyped and genotyped and 85 Earliglow $\hbox {S}_1$ and 77 61S016P006 $S_2$ individuals were phenotyped. Nine-month-old $\hbox {S}_1$ or $\hbox {S}_2$ plants started as seedlings and asexually multiplied bare-root plants of the parents were artificially inoculated with AMP132, transplanted to the field in March 2018, and visually phenotyped weekly for six to 11 weeks post-inoculation.

The 12C089P002 $\times$ PI602575 ($n = 76$), PI552277 $\times$ 12C089P002 ($n = 111$), and PI612569 $\times$ 12C089P002 ($n = 83$) full-sib families, 17C327P010 $\hbox {S}_1$ ($n = 126$) family, and parents of these families were screened for resistance to AMP132 in greenhouse experiments at UC Davis. Two- to four-month-old seedlings of the progeny and bare-root plants of the parents were artificially inoculated with AMP132 and planted in February 2019 (17C327P010 $\hbox {S}_1$), June 2019 (PI552277 $\times$ 12C089P002, PI612569 $\times$ 12C089P002), or November 2019 (12C089P002 $\times$ PI602575) into 10.2 $\times$ 10.2 $\times$ 15.2 cm plastic pots filled with 3 parts coir : 1 part perlite and phenotyped weekly for six to 12 weeks post-inoculation. Four uninoculated and four inoculated single-plant replicates of the parents were arranged in completely randomized experiment designs. The plants were irrigated with a dilute nutrient solution as needed to maintain adequate soil moisture. The 12C089P002 $\times$ PI602575 and PI552277 $\times$ 12C089P002 populations were genotyped with a 50K Axiom SNP array (Hardigan et al. 2020).

### Race 2 resistance screening experiments

We screened a host differential panel ($n = 144$ individuals) for resistance to the MAFF727510 isolate of Fof race 2 in a growth chamber at the UC Davis Controlled Environment Facility in 2018-19. Two single-plant replicates/individual were arranged in a randomized complete block experiment design. The entire experiment was repeated twice, resulting in four clonal replications/individual. The bare-root plants for these experiments were produced in high-elevation nurseries, preserved in cold storage, artificially inoculated with the MAFF727510 isolate, transplanted into 10.2 $\times$ 10.2 $\times$ 15.2 cm plastic pots filled with a 4 parts sphagnum peat moss : 1 part perlite (Sunshine Mix #1; Sun Gro Horticulture, Agawam, MA), and phenotyped weekly for six to 12 weeks post-inoculation. The plants were grown under a 12-hour photoperiod with a 20 °C night temperature and 28 °C day temperature and irrigated with a dilute nutrient solution as needed to maintain adequate soil moisture. Because these experiments utilized a non-California isolate of the pathogen, the experiments were quarantined and conducted in compliance with federally-mandated biosafety regulations (https://www.aphis.usda.gov/aphis/home/).

### SNP genotyping

DNA was isolated from newly emerged leaves harvested from field grown plants using a previously described protocol (Pincot et al. 2020). Leaf samples were placed into 1.5 ml tubes or coin envelopes and freeze-dried in a Benchtop Pro (VirTis SP Scientific, Stone Bridge, NY). Approximately 0.2 g of dried leaf tissue/sample was placed into wells of 2.0 ml 96-well deep-well plates. Tissue samples were ground using stainless steel beads in a Mini 1600 (SPEX Sample Prep, Metuchen, NJ). Genomic DNA (gDNA) was extracted from powdered leaf samples using the E-Z 96®Plant DNA Kit (Omega Bio-Tek, Norcross, GA, USA) according to the manufacturer’s instructions. To enhance the DNA quality and yield and reduce polysaccharide carry-through, the protocol was modified by adding Proteinase K to the lysis buffer to a final concentration of 0.2 mg/ml and extending lysis incubation to 45 min at 65 °C. Once the lysate separated from the cellular debris, RNA was removed by adding RNase A. The mixture was incubated at room temperature for 5 min before a final spin down. To ensure high DNA yields, the sample was incubated at 65 °C for 5 min following the addition of elution buffer. DNA quantification was performed using Quantiflor dye (Promega, Madison, WI) on a Synergy HTX (Biotek, Winooski, VT).

The individuals phenotyped in these studies were genotyped with either 50K or 850K Axiom® SNP arrays (Hardigan et al. 2020). SNP markers on the 50K Axiom array are a subset of those on the 850K Axiom array. The probe DNA sequences for SNP markers on both arrays were previously physically anchored to the 'Camarosa' and 'Royal Royce' reference genomes (Edger et al. 2019; Hardigan et al. 2020, 2021a). The 'Camarosa' genome assembly has been deposited in the Genome Database for the Rosaceae (https://www.rosaceae.org/species/fragaria_x_ananassa/genome_v1.0.a1) and Phytozome (https://phytozome-next.jgi.doe.gov/info/Fxananassa_v1_0_a1). The 'Royal Royce' genome assembly has been deposited in the Genome Database for the Rosaceae (https://www.rosaceae.org/Analysis/12335030) and Phytozome (https://phytozome-next.jgi.doe.gov/info/FxananassaRoyalRoyce_v1_0). The assemblies for each 'Royal Royce' haplotype have been deposited in a Dryad repository (https://doi.org/10.25338/B8TP7G). The physical addresses for the SNP markers are provided in our online resources (https://doi.org/10.25338/B86057). We utilized both reference genomes as needed to cross-check and compare statistical findings and search genome annotations. The results presented in this paper utilized the haplotype-resolved 'Royal Royce' reference genome FaRR1 (Hardigan et al. 2021b) unless otherwise noted. SNP genotypes were called using the Affymetrix Axiom Suite (v1.1.1.66). Samples with call rates exceeding 89-93% were included in genetic analyses.

The haplotypes for 71 50K Axiom array-genotyped SNPs (Hardigan et al. 2020) within a 1.60 Mb haploblock on chromosome 2B (Mb 0.21 to 1.81) were imputed and phased using BEAGLE software version 5.3 (Browning et al. 2018, 2021) for 651 individuals phenotyped for resistance to Fusarium wilt race 1. The individuals were classified as resistant ($1 \le \bar{y} \le 2$) or susceptible ($2 < \bar{y} \le 5$), where $\bar{y}$ is the estimated marginal mean (EMM) for resistance score (*y*) calculated from replicates. We ran BEAGLE with 10 burn-in iterations for imputation and 25 subsequent iterations for phasing on sliding windows of 5 Mb with a window overlap of 2 Mb.

### Statistical analyses of germplasm screening experiments

The R package *lme4* was used for linear mixed model (LMM) analyses of the germplasm screening experiments (Bates et al. 2015). LMMs for square lattice experiment designs were analyzed with entries as fixed effects and incomplete blocks, complete blocks, years, entries $\times$ years, and residuals as random effects (Hinkelmann and Kempthorne 1994). LMMs for randomized complete block experiment designs (ignoring incomplete blocks) were analyzed in parallel to estimate the relative efficiency of the square lattice to the randomized complete block experiment designs (Hinkelmann and Kempthorne 1994). We did not observe an increase in efficiency by using incomplete blocks; hence, the statistics reported throughout this paper were estimated using LMMs for randomized complete block experiment designs (Hinkelmann and Kempthorne 1994). Estimated marginal means (EMMs) for entries were estimated using the R package *emmeans* (Lenth 2017, 2021). Variance components for random effects were estimated using REML (Bates et al. 2015). To estimate broad-sense heritability on a clone-mean basis ($\bar{H}^2 = \bar{\sigma }^2_{G} /\bar{\sigma }^2_P$), analyses were repeated with entries as random effects, where $\bar{\sigma }^2_G$ is the among entry variance, $\bar{\sigma }^2_P = \bar{\sigma }^2_G + \bar{\sigma }^2_{G \times T}/t + \bar{\sigma }^2_E/rt$ is the phenotypic variance on a clone-mean basis, $\bar{\sigma }^2_{G \times T}$ is the entry $\times$ year variance, $\bar{\sigma }^2_E$ is the residual variance, *t* is the number of years, and *r* is the harmonic mean number of replications. Our experiments were designed with four replications/entry; however, because of the random loss of plants (experimental units), *r* was 3.4 in the 2016-17 field experiment, 4.0 in the 2017-18 field experiment, 4.0 in the 2018-19 growth chamber experiment (for race 1 screening), and 3.7 in the 2018-19 growth chamber experiment (for race 2 screening).

### Genome-wide association study

Genome-wide assocation study (GWAS) analyses were carried out to search for the segregation of loci affecting resistance Fusarium wilt races 1 and 2 among individuals genotyped with either the 50K or 850K Axiom SNP array (Hardigan et al. 2020). GWAS analyses were applied to estimated marginal means (EMMs) for resistance phenotypes using physical positions of SNP markers in the 'Camarosa' and 'Royal Royce' reference genomes (Edger et al. 2019; Hardigan et al. 2021b). SNP marker genotypes were coded 1 for AA homozygotes, 0 for heterozygotes, and -1 for aa homozygotes, where A and a are the two SNP alleles. GWAS analyses were performed using the *GWAS* function in the R package *rrBLUP*. The genomic relationship matrix (GRM, K) was estimated from SNP marker genotypes for each population using the *rrBLUP* *A.mat()* function (VanRaden 2008; Endelman 2011). The genetic structure of the GWAS population was investigated using hierarchical clustering and principal components analysis of the GRM as described by Crossa et al. (2014). To correct for population structure and genetic relatedness, a Q + K linear mixed model was used where Q is the population stratification structure matrix and K is the GRM (Yu et al. 2006; Kang et al. 2008). The first three principal components from eigenvalue decomposition of the GRM were incorporated into the Q + K model. Bonferroni-corrected significance thresholds were calculated for testing the hypothesis of the presence or absence of a significant effect. GWAS was repeated in the California population by fitting a SNP marker (AX-184226354) in LD with *FW1* as a fixed effect using the *rrBLUP::GWAS()* function (Endelman 2011).

### Genetic mapping

SNP markers with $\le 5\%$ missing data, high quality codominant genotypic clusters, progeny genotypes concordant with parent gentoypes, and non-distorted segregation ratios (*p* < 0.01) were utilized for genetic and quantitative trait locus (QTL) mapping analyses. The linkage phases of the SNP markers were not known *a priori*. The arbitrarily coded SNPs in the original data were in mixed coupling and repulsion linkage phases. The linkage phases of the SNP markers were ascertained using pair-wise recombination frequency estimates, and recoded so that the 100% of the SNP markers were in coupling linkage phase. This was only necessary in the $\hbox {S}_1$ populations. The recoded SNP markers were genetically mapped in $\hbox {S}_1$ populations using phase-known $\hbox {F}_2$ mapping functions. SNP markers were genetically mapped in full-sib populations using phase-known backcross mapping functions from the subset of SNPs that were heterozygous in the resistant parent and homozygous in the susceptible parent. Genetic maps were constructed using the R packages *onemap* and *BatchMap* (Margarido et al. 2007; Schiffthaler et al. 2017) and custom PERL scripts for binning co-segregating SNP markers, calculating pairwise recombination frequencies, and grouping markers using LOD threshold of 10 and maximum recombination frequency threshold of 0.05. The custom PERL scripts are available in the Dryad repository for this paper (https://doi.org/10.25338/B86057). Linkage groups were aligned and assigned to chromosomes using inter-group linkage disequilibrium statistics and percent-identity against the reference genome (Edger et al. 2019; Hardigan et al. 2021b). Marker orders and genetic distances were estimated in parallel using the RECORD algorithm in *Batchmap* with a 25-marker window, window overlap of 15 markers, and ripple window of six markers (Van Os et al. 2005; Schiffthaler et al. 2017). For smaller linkage groups, the window size was reduced incrementally by five to ensure at least two overlapping windows. We used the *checkAlleles*, *calc.errorlod*, and *top.errorlod* functions of the R package *qtl* (Lincoln and Lander 1992; Broman et al. 2003) and custom R scripts to identify and eliminate spurious SNP markers and successively reconstruct linkage groups as described by Phansak et al. (2016). Genetic distances (cM) were estimated from recombination frequencies using the Kosambi mapping function (Kosambi 1943).

### QTL mapping

We applied two approaches to scan the genome for the segregation of quantitative trait loci affecting resistance to Fusarium wilt race 1 in $\hbox {S}_1$ or full-sib populations genotyped with the 50K Axiom SNP array (Online Resource 2). First, the effects of individual SNP marker loci were estimated using single marker regression as implemented in the R package *qtl* (Broman et al. 2003). The test statistics for each SNP marker locus were plotted against physical positions in the 'Royal Royce' reference genome (Hardigan et al. 2021b). Second, QTL effects were estimated using Haley-Knott interval mapping as implemented in the R package *qtl* (Haley and Knott 1992; Broman et al. 2003). These analyses used the positions of markers estimated by *de novo* genetic mapping (Online Resources 2, 3). Genome-wide significance thresholds (*p* = 0.05) were calculated by permutation testing with 2,000 permutations (Sen and Churchill 2001). We estimated 95% Bayes confidence intervals for QTL using the *bayes.int* function (Broman and Sen 2009). The percentage of the phenotypic variance (PVE) explained by a SNP marker locus was estimated using the bias-corrected average semivariance method described by Feldmann et al. (2021), where PVE = $\hat{\sigma }^2_M/\hat{\sigma }^2_P \times 100$, $\hat{\sigma }^2_M$ is a bias-corrected REML estimate of the fraction of the genetic variance explained by a SNP marker locus, and $\hat{\sigma }^2_P$ is a REML estimate of the phenotypic variance for resistance to race 1. For statistical analyses of SNP marker loci segregating in $\hbox {S}_1$ populations, the additive effect ($\hat{a}$) was estimated by $\hat{a} = [\bar{y}_{AA}-\bar{y}_{aa}]/2$, the dominance effect was estimated by $\hat{d} = \bar{y}_{Aa} - [\bar{y}_{AA} + \bar{y}_{aa}]/2$), and the degree of dominance was estimated by $\hat{d}/\hat{a}$, where $\bar{y}_{aa}$, $\bar{y}_{Aa}$, and $\bar{y}_{AA}$ are the respective estimated marginal means (EMMs) for individuals with *aa*, *Aa*, and *AA* SNP marker genotypes, the *a* was transmitted by the susceptible parent, and the *A* allele was transmitted by the resistant parent. For statistical analyses of SNP marker loci segregating in full-sib populations, effects were estimated the difference between EMMs for heterozygous (*Aa*) and homozygous (*aa*) individuals ($\bar{y}_{Aa}-\bar{y}_{aa}$).

### KASP marker development

Kompetitive allele specific primer (KASP) markers were developed for SNPs predicted to be tightly linked to the Fusarium wilt *R*-genes identified in our studies (Semagn et al. 2014; https://www.biosearchtech.com/support/education/kasp-genotyping-reagents). KASP primers were designed using PolyOligo (Ledda et al. 2020; https://github.com/MirkoLedda/polyoligo) with the 'Camarosa' reference genome (Hardigan et al. 2020, 2021b). We used default PolyOligo design parameters and only tested primers for KASP markers with heuristic quality scores $\ge 7$ on a 1 to 10 scale. KASP markers were tested by screening a diverse sample of race 1 resistant and susceptible individuals ($n = 186$) and mapping population progeny. To assess the prediction accuracies of KASP markers, we estimated the concordance between marker genotypes and dominant *R*-gene genotypes inferred from resistance phentoypes ($A\_$ for resistant and *aa* for susceptible individuals). KASP-SNP marker genotyping was outsourced to LGC Biosearch Technologies (Hoddesdon, United Kingdom; https://www.biosearchtech.com/support/education/kasp-genotyping-reagents/kasp-overview). The physical locations of SNPs in the 'Camarosa' and 'Royal Royce' reference genomes, oligonucletoide primer sequences, and other supporting data for KASP markers are compiled in Online Resource 3.

## Results

### Resistance to Fusarium wilt race 1 is widespread in natural and domesticated populations

**Fig. 1:**
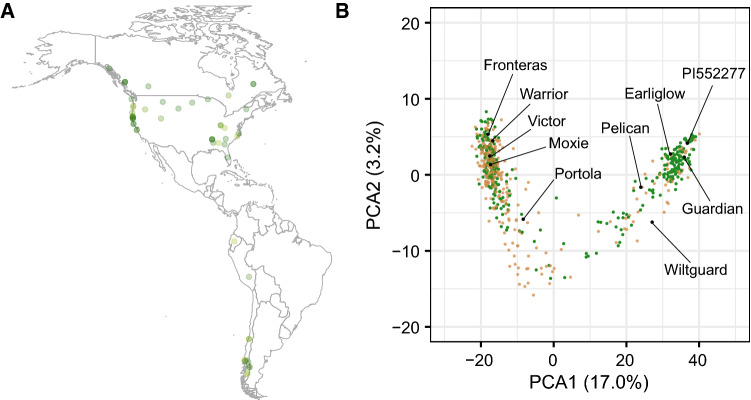
Genetic diversity of octoploid germplasm accessions screened for resistance to Fusarium wilt race 1. **A** Geographic distribution (latitude and longitude coordinates) for 27 *F. chiloensis* and 21 *F. virginiana* ecotypes classified as resistant ($1.0 \le \bar{y} \le 2.0$) to the AMP132 isolate of *F. oxysporum* f. sp. *fragariae* race 1, where $\bar{y}$ is the estimated marginal mean (EMM) for disease ratings over replications and years (see Online Resource 1 for EMMs and other supporting data). The opaqueness of the points increases as resistance increases (as the EMM decreases). **B** Genetic diversity among 11 *F. chiloensis*, 21 *F. virginiana*, and 608 *F.* $\times$ *ananassa* individuals estimated from the genotypes of 31,212 SNP marker loci assayed with a 50K Axiom SNP array (Hardigan et al. 2020). The first two principal scores from a principal component analysis of the $640 \times 640$ genomic relationship matrix are displayed with resistant individuals ($1.0 < \bar{y} \le 2.0$) shown in green and susceptible individuals ($2.0 < \bar{y} \le 5.0$) shown in light brown

Two-thirds of the octoploid strawberry germplasm accessions (ecotypes, cultivars, and other clonally preserved individuals) screened for resistance to Fusarium wilt race 1 in the present study (226/344 = 0.66) had disease symptom ratings in the resistant range ($1.0 \le \bar{y} \le 2.0$), where $\bar{y}$ is the estimated marginal mean (EMM) among replicates and years, $y = 1$ plants were symptomless, and $y = 2$ plants were nearly symptomless (Fig. 1; Online Resource 1). The other one-third (118/344 = 0.34) had disease symptom ratings in the moderately to highly susceptible range ($2 < \bar{y} \le 5$). The severity of symptoms (e.g., chlorosis and wilting) increased as $\bar{y}$ increased on our ordinal scale (plants with scores of five were killed by the pathogen). The race 1 resistance phenotypes observed among resistant and susceptible checks in the present study were consistent with those previously reported (Pincot et al. 2018; Online Resource 1). The repeatability of race 1 resistance phenotypes among clonal replicates of resistant and susceptible checks was 0.81.

One-fourth of the individuals (81/344 = 0.24) screened in the present study were symptomless, classified as highly resistant ($1.0 \le \bar{y} \le 1.5$), and appeared to be immune to AMP132 infections (Fig. 1; Online Resource 1). This confirmed our suspicion that resistance to race 1 was widespread in natural and domesticated populations of octoploid strawberry. We suspected this because the only ‘non-California’ individuals screened in our previous study (Earliglow and Guardian) were highly resistant to AMP132 infection, had non-*FW1* SNP marker haplotypes, and were presumed to carry novel *R*-genes (Pincot et al. 2018). Moreover, the individuals screened in the present study were more diverse than those previously screened from the California population (Fig. 1; Hardigan et al. 2021a; Pincot et al. 2021).

Slightly more than half of the *F. chiloensis* and *F. virginiana* ecotypes (55/104 = 0.53) screened for resistance to race 1 in the present study were classified as resistant ($1.0 \le \bar{y} \le 2.0$; Online Resource 1). We did not observe geographic or phylogenetic trends—highly resistant ecotypes were found throughout the natural geographic ranges of both species (Fig. 1; Online Resource 1). Eleven out of 62 *F. chiloensis* and 12 out of 40 *F. virginiana* ecotypes were classified as highly resistant ($1.0 \le \bar{y} \le 1.5$). Moreover, highly resistant ecotypes were identified for each of the seven subspecies of *F. chiloensis* and *F. virginiana* apart from one ecotype of *F. virginiana* subsp. *platypetala*, which was nevertheless classified as resistant ($\bar{y} = 1.9$). We did not screen ecotypes of *F. chiloensis* subsp. *sandwichensis*, the subspecies found in Hawaii (Staudt 1989), because none were available when our study was undertaken.

**Fig. 2:**
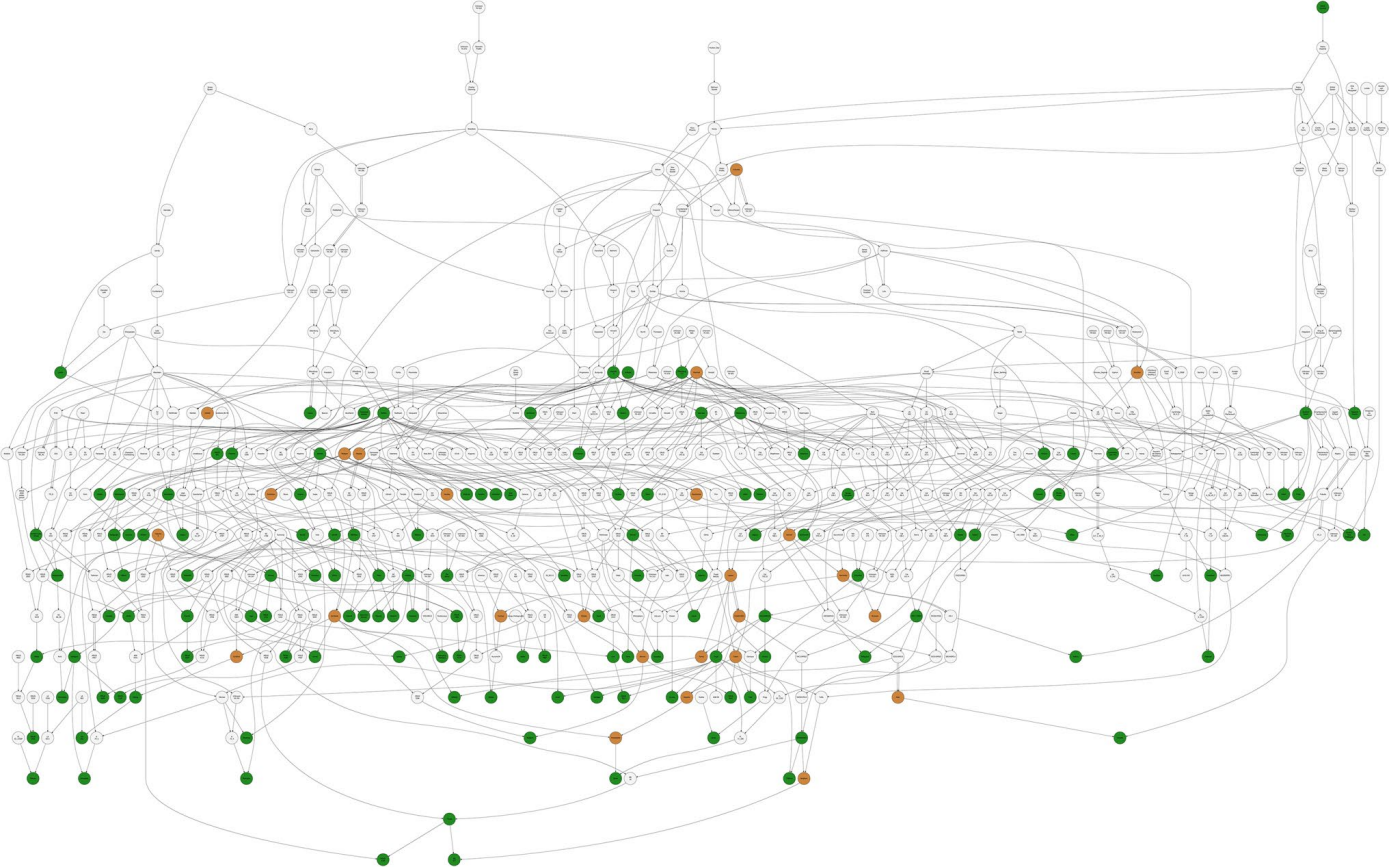
Pedigree network for Fusarium wilt race 1 resistant strawberry germplasm accessions. Pedigrees are displayed for 142 *F.* $\times$ *ananassa* individuals. The individuals with green or light brown nodes were screened for resistance to the AMP132 isolate of Fusarium wilt race 1. Green nodes identify resistant individuals ($1.0 \le \bar{y} \le 2.0$) and light brown nodes identify susceptible individuals ($2.0 < \bar{y} \le 5.0$), where $\bar{y}$ is the estimated marginal mean for resistance to race 1 over replications and years. The race 1 resistance phenotypes of ancestors with light gray nodes are unknown

Approximately two-thirds of the *F.* $\times$ *ananassa* individuals screened in the present study (161/227 = 0.71) were resistant ($1.0 < \bar{y} \le 2.0$) to AMP132 infection (Fig. 2; Online Resource 1). Of these, 20 originated in the California (CA) population and were either known or predicted to carry *FW1* from SNP marker haplotypes (Pincot et al. 2018; Online Resource 4). Other than Wiltguard (the only non-*FW1* resistant individual from the CA population), the other 141 are heirloom and historically important *F.* $\times$ *ananassa* individuals originating in North America, Europe, and Japan between 1880 and 1987 (Fig. 2; Online Resource 1; Online Resource 4).

We extracted the pedigree records for Wiltguard (an outlier in the CA population) and the 141 non-CA individuals from the database described by Pincot et al. (2021) to show that the genetic relationships among most of these individuals and their ascendants were complex and intertwined (Fig. 2; Online Resource 5). Of 603 individuals in the pedigree network, 101 were identified to be founders (have unknown parents) and 180 were phenotyped for resistance to Fusarium wilt race 1 (Fig. 2; Online Resource 5). Twenty-one individuals either lacked pedigree records or only had a single-generation of pedigree records with one or both parents known (pedigree network orphans). The orphans are identified in the pedigree database (Online Resource 5) but not shown in Fig. 2, which displays interconnections among the other 120 non-UC individuals and Wiltguard (the non-*FW1* outlier in the UC population). The pedigree network diagram and database show that the alleles found in these resistant individuals have flowed through several common ancestors (Fig. 2). Although the presence of multiple *R*-genes cannot be ruled out—partly because some of the individuals are orphans or extinct and phenotypes are only known for a subset of the ascendants of resistant individuals—there is a high probability that the number of unique alleles is small and that many of the *R*-alleles found in cultivars worldwide are identical-by-descent (Online Resource 5). Our studies targeted two heirloom cultivars (Wiltguard and Guardian) that share three resistant common ancestors (White Carolina, Howard 17, and Blakemore), had unique haplotypes for SNPs in LD with *FW1*, were genetically distant from one another and individuals in the California population, and were predicted to have a high probability of carrying novel *R*-genes (Figs. 1, 2; Online Resource 6). The pedigrees for these cultivars are shown in Online Resource 7 (Figs. S1 and S2).

### Screening global diversity uncovers several sources of resistance to Fusarium wilt race 2

Selection of individuals for constructing a host differential panel ($n = 144$) was informed by insights gained from screening public germplasm collections for resistance to race 1 ($n = 981$ accessions) and insights gained from population structure analyses in strawberry (Hardigan et al. 2020, 2021a; Pincot et al. 2021). The host differential panel was assembled to maximize the probability of differentiating races and identifying sources of resistance to different Fof isolates (Online Resource 1). The phenotypes for resistance to AMP132, MAFF727510, and four other Fof isolates were previously reported for 25 octoploid individuals on the host differential panel: one *F. chiloensis* ecotype, one *F. viriginiana* ecotype, and 23 *F.* $\times$ *ananassa* individuals (Pincot et al. 2018; Henry et al. 2021). MAFF727510 is an Fof race 2 isolate found in Japan (Henry et al. 2021). To broaden insights into the frequency and distribution of race 2 resistance sources, the phenotypes for resistance to MAFF727510 are reported here for an additional 116 individuals: 10 *F. chiloensis* ecotypes, 16 *F. viriginiana* ecotypes, and 93 *F.* $\times$ *ananassa* individuals (Online Resource 1). The latter included cultivars and other individuals selected to broadly sample allelic diversity in California and non-California populations worldwide. Similarly, the ecotypes were selected to sample allelic diversity across the natural ranges of *F. chiloensis* and *F. viriginiana*.

The race 1 and 2 resistance phenotypes observed in these studies were highly repeatable: estimates of broad-sense heritability were $\hat{H}^2 = 0.98$ for resistance to AMP132 (race 1) and $\hat{H}^2 = 0.91$ for resistance to MAFF727510 (race 2). Forty-one individuals on the host differential panel (28.5%) were classified as resistant ($1.0 < \bar{y} \le 2.0$) to race 2 (Online Resource 1). Thirty-four of these individuals were symptomless and classified as highly resistant ($1.0 < \bar{y} \le 1.5$), and 34 of the race 2 resistant accessions (87.8%) were resistant to race 1 (Online Resource 1). Of the 78 *F.* $\times$ *ananassa* individuals from the California population, only four (5.1%) were resistant to MAFF727510. Conversely, of the 38 *F.* $\times$ *ananassa* individuals from the non-California population, 21 (55%) were resistant to MAFF727510. Slightly more than half of the *F. chiloensis* and *F. virginiana* ecotypes were resistant to race 2 (16/28 = 57%), which was comparable to the frequency observed for race 1 resistance (55/104 = 53%; Fig. 1; Online Resource 1). Three individuals on the host differential panel (61S016P006, Earlimiss, and Earliglow) were resistant to every Fof isolate tested from California, Japan, Australia, and Spain (Online Resource 1; Henry et al. 2021). According to historical breeding records (Pincot et al. 2021), Royce S. Bringhurst developed 61S016P006 (PI551676), an $\hbox {S}_1$ descendant of 43C001P036, by selecting for resistance to Verticillium wilt in Davis, California, nearly a half century before Fusarium wilt was discovered in California (Koike et al. 2009; Koike and Gordon 2015).

Using the host differential panel as the study population and 50K Axiom SNP array genotypes, we searched the genome for associations between SNP marker loci and race 2 resistance phenotypes. Statistically significant GWAS signals for loci affecting resistance to race 2 were not observed (Online Resource 7 Fig. S3; Online Resource 8). We repeated this analysis with the host differential panel using race 1 resistance phenotypes and reproduced the strong GWAS signal associated with the segregation of *FW1* in the California population (Online Resource 7 Fig. S3; Online Resource 8). The absence of a significant GWAS signal for race 2 resistance has several possible explanations. First, resistance to race 2 might not be governed by gene-for-gene resistance. Second, resistance to race 2 could be governed by gene-for-gene resistance but undetectable in a highly diverse population where multiple alleles and loci are segregating and the resistant alleles are uncommon. Those alleles, however, could almost certainly be uncovered and identified by forward genetic analyses of segregating populations developed from crosses between resistant and susceptible parents, as described below for the race 1 resistance loci identified in the present study. Third, the sample size ($n = 144$) may have been insufficient to detect the presence of gene-for-gene resistance to race 2. This seems unlikely because *R*-genes have large effects, and we have consistently observed strong GWAS signals for *FW1* in small samples of California population individuals, including the host differential panel (Online Resource 7 Fig. S3; Online Resource 8).

**Table 1:** Statistics for SNP markers associated with genes conferring resistance to Fusarium wilt race 1

Population${}^{\mathrm{a}}$	*n*	Locus${}^{\mathrm{b}}$	Chr${}^{\mathrm{c}}$	SNP Marker${}^{\mathrm{d}}$	PVE${}^{\mathrm{e}}$		EMMs and Contrasts${}^{\mathrm{f}}$					$\hat{d}/\hat{a}$
						$\bar{y}_{AA}$	$\bar{y}_{Aa}$	$\bar{y}_{aa}$	$\bar{y}_{Aa}-\bar{y}_{aa}$	$\hat{a}$	$\hat{d}$	
Fronteras $\hbox {S}_1$	92	*FW1*	2B	AX-166521396	77.6	1.25	1.24	2.94	–	–0.85	–0.86	1.01
Portola $\hbox {S}_1$	92	*FW1*	2B	AX-166521396	75.8	1.07	1.25	3.31	–	–1.12	–0.94	0.84
Guardian $\hbox {S}_1$	99	*FW2*	2B	AX-184486400	76.4	1.16	1.40	4.45	–	–1.65	–1.41	0.85
Wiltguard $\hbox {S}_1$	98	*FW3*	1A	AX-123363542	22.6	1.53	1.76	2.87	–	–0.66	–0.44	0.66
PI552277 $\times$ 12C089P002	111	*FW4*	6B	AX-184298748	42.9	–	1.69	4.00	–1.15	–	–	–
12C089P002 $\times$ PI602575	76	*FW5*	2B	AX-184226354	84.1	–	1.06	4.29	–1.61	–	–	–

${}^{\mathrm{a}}$ $\hbox {S}_1$ families were produced by self-pollinating resistant individuals heterozygous for Fusarium wilt *R*-genes. Subsequent to phasing the SNP markers, standard phase-known $\hbox {F}_2$ statistical methods were applied in genetic analyses of $\hbox {S}_1$ families. Full-sib (FS) families were produced by crossing a homozygous susceptible parent (12C089P002) with heterozygous resistant parents (PI552277 and PI602575). Standard backcross statistical methods were applied in genetic analyses of full-sib families using SNP markers that were heterozygous (*Aa*) in the resistant parent and homozygous (*aa*) in the susceptible parent, where *A* is the allele transmitted by the resistant parent and *a* is the allele transmitted by the susceptible parent. Statistics shown for the Fronteras and Portola $\hbox {S}_1$ populations are adapted from Pincot et al. (2018)

${}^{\mathrm{b}}$ *FW1* was previously identified by Pincot et al. (2018)

${}^{\mathrm{c}}$ Chromosome (Chr) numbers follow the nomenclature proposed by Hardigan et al. (2021a) and applied in the annotation of the 'Royal Royce' genome (Hardigan et al. 2021b)

${}^{\mathrm{d}}$ SNP marker identification number on the 50K Axiom SNP array (Hardigan et al. 2020)

${}^{\mathrm{e}}$ The percentage of the phenotypic variance (PVE = $\hat{\sigma }^2_M/\hat{\sigma }^2_P \times 100$) explained by a SNP marker associated with a resistance locus was estimated using the average semivariance method of Feldmann et al. (2021), where $\hat{\sigma }^2_M$ is a bias-corrected REML estimate of the fraction of the genetic variance explained by a SNP marker locus and $\hat{\sigma }^2_P$ is a REML estimate of the phenotypic variance for resistance to race 1

${}^{\mathrm{f}}$ $\bar{y}_{aa}$, $\bar{y}_{Aa}$, and $\bar{y}_{AA}$ are the estimated marginal means (EMMs) for individuals with *aa*, *Aa*, and *AA* SNP marker genotypes for a SNP marker locus (*A*) genotyped in a segregating population, where the *a* allele was transmitted by the susceptible parent and the *A* allele was transmitted by the resistant parent. For statistical analyses of SNP marker loci segregating in $\hbox {S}_1$ populations, the additive effect ($\hat{a}$) was estimated by $\hat{a} = [\bar{y}_{AA}-\bar{y}_{aa}]/2$, the dominance effect was estimated by $\hat{d} = \bar{y}_{Aa} - [\bar{y}_{AA} + \bar{y}_{aa}]/2$), and the degree of dominance was estimated by $\hat{d}/\hat{a}$. For statistical analyses of SNP marker loci segregating in full-sib populations, effects were estimated from contrasts between EMMs for heterozygous (*Aa*) and homozygous (*aa*) individuals (shown in the $\hat{a}$ column). The $\bar{y}_{Aa} - \bar{y}_{aa}$ contrast estimates the additive effect of the SNP marker locus only when $|\hat{d}/\hat{a}| = 1$ (when the *A* allele is dominant)

**Table 2:** Genomic locations and prediction accuracy statistics for KASP-SNP markers associated with genes conferring resistance to Fusarium wilt race 1

KASP marker name${}^{\mathrm{a}}$	Locus	Chr${}^{\mathrm{b}}$	FaCA1 genome position (bp)${}^{\mathrm{c}}$	FaRR1 genome position (bp)${}^{\mathrm{d}}$	SNP (R/S)${}^{\mathrm{e}}$	Axiom Array SNP Marker	Discovery population prediction accuracy (%)${}^{\mathrm{f}}$	CA population prediction accuracy (%)${}^{\mathrm{g}}$	Non-CA population prediction accuracy (%)${}^{\mathrm{h}}$
FW1_K7	*FW1*	2B	20,343	432,840	T/G	AX-184585165	98.8	97.5	35.9
FW1_K6	*FW1*	2B	31,131	443,570	T/G	AX-184950786	98.7	97.5	24.6
FW1_K3	*FW1*	2B	373,024	804,139	G/T	AX-184624236	98.8	95.0	43.8
FW2_K3	*FW2*	2B	505,395	951,437	C/A	AX-184495646	97.5	62.8	75.8
FW2_K4	*FW2*	2B	1,174,541	NA	T/C	AX-184456942	95.0	94.0	72.3
FW3_K4	*FW3*	1A	NA	6,781,226	A/G	AX-166511067	97.1	41.8	67.2
FW3_K1	*FW3*	1A	NA	6,878,550	A/T	AX-123363542	100.0	45.1	35.6
FW3_K3	*FW3*	1A	6,547,713	7,028,803	C/T	AX-184165918	100.0	34.6	38.1
FW3_K5	*FW3*	1A	6,574,293	7,054,363	A/G	AX-184204436	100.0	45.0	36.5
FW4_K2	*FW4*	6B	22,154,455	13,987,603	A/G	AX-184854002	97.4	56.4	77.4
FW4_K1	*FW4*	6B	21,416,161	14,800,900	T/G	AX-184298748	97.5	22.9	76.9
FW4_K5	*FW4*	6B	22,542,704	15,815,205	C/T	AX-184366576	95.0	56.8	51.7
FW4_K3	*FW4*	6B	19,888,153	16,348,827	T/C	AX-184030353	95.0	26.8	76.9
FW5_K4	*FW5*	2B	1,491,222	1,732,659	G/T	AX-184348754	90.0	45.7	70.3

${}^{\mathrm{a}}$KASP-SNP markers are identified by the Fusarium wilt resistance locus and an alphanumeric suffix starting with a K and ending with an integer

${}^{\mathrm{b}}$Chromosome (Chr) numbers follow the nomenclature proposed by Hardigan et al. (2021a)

${}^{\mathrm{c}}$Physical position of the SNP in the 'Camarosa' reference genome (Edger et al. 2019)

${}^{\mathrm{d}}$Physical position of the SNP in the 'Royal Royce' reference genome (Hardigan et al. 2021b)

${}^{\mathrm{e}}$The SNP allele transmitted by the resistant (R) parent is shown to the left, whereas the SNP allele transmitted by the susceptible (S) parent is shown to the right of the slash

${}^{\mathrm{f}}$Prediction accuracy statistics for KASP-SNP markers genotyped in a random sample of 40 individuals within each of the original segregating populations developed to discover the Fusarium wilt resistance loci. The prediction accuracy estimates shown here are the frequencies with which SNP marker genotypes correctly identified resistant and susceptible individuals in a particular study population

${}^{\mathrm{g}}$Prediction accuracy statistics for KASP-SNP markers genotyped among 86 individuals in the California population

${}^{\mathrm{h}}$Prediction accuracy statistics for KASP-SNP markers genotyped among 37 non-California *F.* $\times$ *ananassa* cultivars, 12 *F. chiloensis* ecotypes, and 17 *F. virginana* ecotypes

**Fig. 3:**
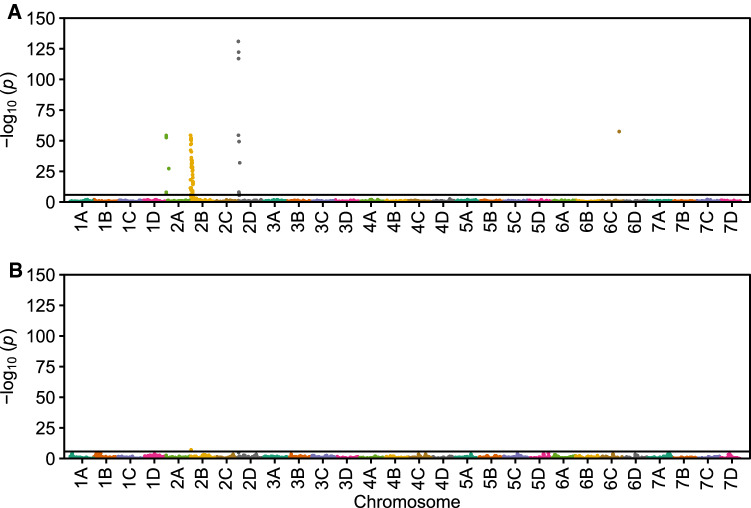
Genome-wide associations between SNP markers and Fusarium wilt resistance phentoypes. Manhattan plots displaying associations between SNP markers and Fusarium wilt race 1 resistance phenotypes observed among California population individuals phenotyped for resistance to the AMP132 isolate of *F. oxysporum* f. sp. *fragariae*. **A** The upper Manhattan plot displays statistics estimated from the resistance phenotypes of 302 individuals genotyped with the 50K Axiom SNP array (Hardigan et al. 2020). The SNP markers were anchored *in silico* to the 'Royal Royce' genome (Hardigan et al. 2021b). **B** The lower Manhattan plot displays statistics estimated from the same data by fitting the the AX-184226354 SNP marker from chromosome 2B as a fixed effect. The horizontal lines identify the Bonferroni-corrected significance thresholds for hypothesis testing (*p* = 1.6 $\times$ 10^-6^)

**Fig. 4:**
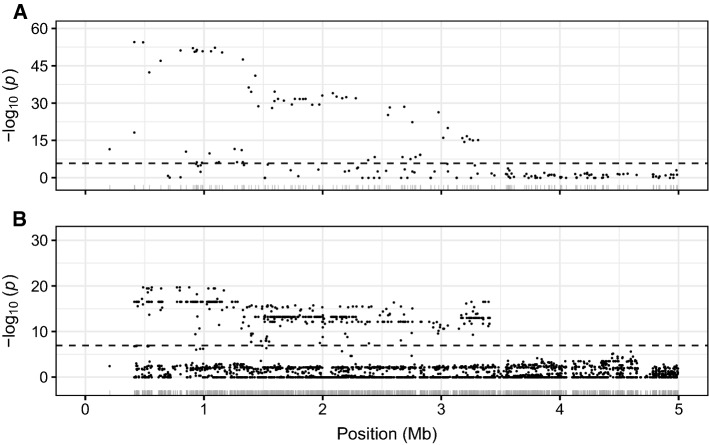
Associations between SNP markers and Fusarium wilt race 1 resistance phenotypes on the upper arm of chromosome 2B. **A** GWAS statistics are shown for the upper 5 Mb haploblock on chromosome 2B from an analysis of race 1 resistance phenotypes among 302 individuals in the California population genotyped with a 50K Axiom SNP array. The individuals in this study were previously phenotyped for resistance using the AMP132 race 1 isolate of *F. oxysporum* f. sp. *fragariae* and predicted to be segregating for *FW*1 (Pincot et al. 2018). The SNP markers were physically mapped to the 'Royal Royce' genome (Hardigan et al. 2021b). Their positions are shown in the rug plot along the x-axis. **B** GWAS statistics are shown for an identical analysis of 54 previously phenotyped individuals in the California population. These individuals were genotyped with an 850K Axiom SNP array

**Fig. 5:**
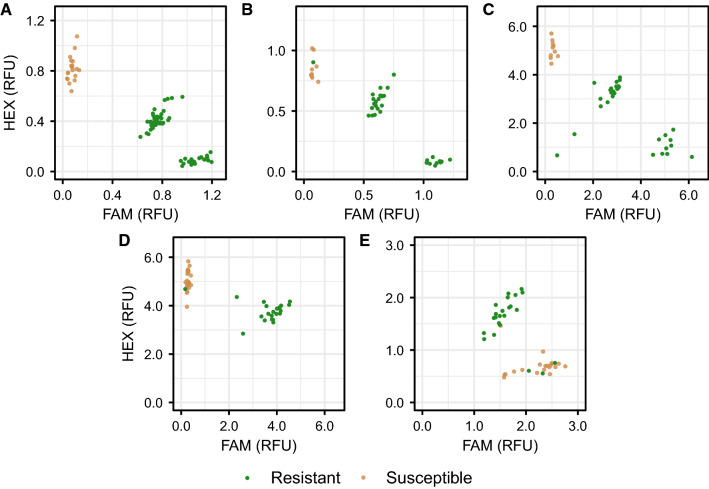
Kompetitive Allele Specific PCR (KASP) markers for single nucleotide polymorphisms in linkage disequilibrium with Fusarium wilt resistance (*FW*) loci in strawberry. Resistant individuals ($1.0 < \bar{y} \le 2.0$) are shown in green, susceptible individuals ($2.0 < \bar{y} \le 5.0$) are shown in brown. FAM and HEX signals are reported in relative fluorescence units (RFUs). Fluorescence intensities were normalized using a passive reference dye (ROX). **A** *FW1_K7* KASP marker genotypes observed in the Portola $\hbox {S}_1$ ($n = 40$) and the Fronteras $\hbox {S}_1$ ($n = 40$) populations for a SNP associated with the *FW1* locus. **B** *FW2_K3* KASP marker genotypes observed in the Guardian $\hbox {S}_1$ population ($n = 40$) for a SNP associated with the *FW2* locus. **C** *FW3_K3* KASP marker genotypes observed in the Wiltguard $\hbox {S}_1$ population ($n = 40$) for a SNP associated with the *FW3* locus. **D** *FW4_K1* KASP marker genotypes observed targeting *FW4* in the PI552277 $\times$ 12C089P002 full-sib population ($n = 40$) for a SNP associated with the *FW4* locus. **E** *FW5_K4* KASP marker genotypes observed in the 12C089P002 $\times$ PI602575 full-sib population ($n = 40$) for a SNP associated with the *FW5* locus

### Association mapping pinpointed the *FW1* locus to a short haploblock on chromosome 2B

The *FW1* locus was previously identified and physically mapped using diploid genome-informed GWAS in a closed UCD or ’California’ population of 565 individuals genotyped with a SNP array populated with diploid genome-anchored SNPs (Pincot et al. 2018). To revisit our original analyses using octoploid genome-informed GWAS, 356 of these individuals were genotyped with either the 50K or 850K Axiom SNP arrays (Figs. 3, 4; Online Resource 7 Fig. S5). This substantially increased the density and uniformity of SNPs in the *FW1* haploblock and facilitated a search for genes with plant defense annotations because the SNP markers on both arrays were physically anchored *in silico* to octoploid reference genomes developed since the original study was reported (Edger et al. 2019; Hardigan et al. 2020, 2021b, a). The physical positions of *FW1*-associated SNPs in the ’Camarosa’ and ‘Royal Royce’ genomes are shown in Online Resource 8.

GWAS and *de novo* genetic mapping pinpointed the location of the *FW1* locus to a near-telomeric haploblock on the upper arm of chromosome 2B spanning approximately 3.3 Mb (Fig. 4; Online Resource 8). The 3.3 Mb haploblock was populated with 1,725 SNP markers from the 50K and 850K Axiom SNP arrays, of which 460 were significantly associated with race 1 resistance phenotypes (Fig. 4; Online Resource 8). SNP markers with the strongest GWAS signals on chromosome 2B were AX-184226354 (0.414 Mb; $-\log _{10}p = 54.6$) and AX-184176344 ($-\log _{10}p = 54.6$) (Fig. 4; Table 1). KASP markers were developed for SNPs predicted by GWAS to be in LD with *FW1* (Table 2). These were genotyped in the Fronteras and Portola $\hbox {S}_1$ populations, shown to be tightly linked to the *FW1* locus on chromosome 2B, and estimated to predict race 1 resistance phenotypes with 98.7 to 98.8% accuracy in the Fronteras and Portola $\hbox {S}_1$ populations and 95.2 to 97.6% accuracy in the California population (Tables 1, 2; Fig. 5).

Even though GWAS signals were observed between resistance phenotypes and SNP markers predicted to reside on homoeologous chromosomes, 84% (68/81) of the 50K and 80% (392/488) of the 850K Axiom array SNP markers with statistically significant GWAS signals were predicted *in silico* to reside on chromosome 2B proximal to the previously genetically mapped *FW1* locus (Figs. 3, 4; Online Resource 7 Figs. S5 and S6; Pincot et al. 2018). Nevertheless, the strongest GWAS signals were observed for SNP markers that had previously been physically assigned *in silico* to chromosome 2D: AX-89872358 ($-\log _{10}p = 130.9$), AX-184098127 ($-\log _{10}p = 122.2$), and AX-184513679 ($-\log _{10}p = 117.0$) on the 50K SNP array and AX-184055143 ($-\log _{10}p = 26.6$) on the 850K SNP array (Fig. 4; Online Resource 7 Fig. S5; Online Resource 8; Hardigan et al. 2020).

The GWAS signals observed on chromosomes other than 2B were almost certainly caused by incorrect *in silico* physical assignments of Axiom SNP array probe DNA sequences to positions in octoploid reference genomes (Fig. 3). This conclusion was supported by several observations and analyses. First, QTL mapping in the Fronteras and Portola $\hbox {S}_1$ populations only uncovered statistically significant signals for race 1 resistance on chromosome 2B (Table 1; Pincot et al. 2018). Second, GWAS was repeated for race 1 resistance phenotypes observed in the California population by fitting AX-184226354 as a fixed effect, then searching the genome for significant GWAS signals—AX-184226354 was the SNP marker on chromosome 2B that was most strongly associated with the *FW1* locus (Tables 1, 2). GWAS with AX-184226354 incorporated as a fixed effect eliminated signals on other chromosomes, in addition to eliminating signals for other tightly linked SNPs on chromosome 2B (Fig. 3B; Online Resource 7 Fig. S5). Hence, fitting and testing multilocus genetic models with SNPs (independent variables) selected from single locus genome-wide searches (initial locus-by-locus GWAS) is a powerful approach for resolving physical address assignment errors, a particular problem in polyploids, and generating more accurate estimates of genetic parameters (Endelman 2011; Feldmann et al. 2021).

A certain percentage of erroneous (off-target) GWAS signals are expected in octoploid strawberry because a small percentage of the short 71-nt DNA probe sequences for Axiom array SNP markers are incorrectly assigned *in silico* to physical positions in the reference genome, e.g., Hardigan et al. (2020) estimated that approximately 74% of QC-passing 850K Axiom SNP array probes could be assigned to the correct homoeolog in the ‘Camarosa’ genome. That percentage was virtually identical to the percentage of Axiom array SNPs with significant GWAS signals on chromosomes other than 2B in our analyses (Online Resources 2 and 8). Genetic mapping of Axiom array SNPs in the present and previous studies have shown that the percentage of Axiom array SNPs with physical positions that were incorrectly assigned *in silico* are found on homoeologous chromosomes (Fig. 3; Online Resource 7 Fig. S5). This GWAS complication is bound to arise in strawberry and other polyploid and paleopolypoid species with complex repetitive DNA landscapes, especially outbred (heterozygous) species with whole genome duplications where homoeologous DNA variation complicates the physical assignment of short DNA sequences to subgenomes (Hardigan et al. 2020). The assignment of highly accurate long-read DNA sequences, by contrast, is straightforward (Hardigan et al. 2021b). The octoploid genome-informed GWAS analyses described here were initiated in 2017 immediately after we assembled the ‘Camarosa’ reference genome (Edger et al. 2019). We have since expanded our understanding of the complexity of the octoploid genome, built superior haplotype-phased genome assemblies (Hardigan et al. 2021b), and unequivocally shown that homologous and homoeologous DNA variation can be differentiated nearly genome-wide in strawberry (Hardigan et al. 2020, 2021a). Our analyses show that false positive and ‘off-target’ GWAS signals that arise because of erroneous physical addresses can typically be identified and rectified by fitting DNA markers associated with causal loci as fixed effects, which is effectively equivalent to fitting a multilocus genetic model in a QTL mapping or candidate gene analysis study using mixed linear models (Feldmann et al. 2021). A preponderance of the erroneous physical addresses are typically going to be found on homoeologous chromosomes, as was the case in our study, and hence could be misconstrued as signals associated with the effects of homoeologous loci.

### Mendelian genetic analyses uncover additional sources of gene-for-gene resistance to Fusarium wilt race 1

The sheer numerical abundance of sources of resistance to Fusarium wilt race 1 in strawberry did not shed light on the diversity of *R*-genes that they might carry, if any, or genetic mechanisms underlying resistance (Figs. 1, 2). Was resistance to race 1 conferred by dominant *R*-genes? How many unique Fusarium wilt *R*-genes exist in wild and domesticated populations of strawberry? To explore these questions, we developed and undertook genetic analyses of $\hbox {S}_1$ populations developed by self-pollinating highly resistant *F.* $\times$ *ananassa* heirloom cultivars (Earliglow, Guardian, and Wiltguard) and individuals (61S016P006 and 17C327P010) and full-sib populations developed by crossing highly resistant ecotypes of *F. chiloensis* subsp. *chiloensis* (PI602575; $\bar{y} = 1.3$), *F. virginiana* subsp. *virginiana* (PI552277; $\bar{y} = 1.0$), and *F. virginiana* subsp. *grayana* (PI612569; $\bar{y} = 1.0$) with a highly susceptible *F.* $\times$ *ananassa* individual (12C089P002; $\bar{y} = 5.0$) (Table 3).

**Table 3:** Goodness-of-fit statistics for mendelian genetic analyses of the segregation of Fusarium wilt race 1 resistance genes

Population	Resistant parent	Resistant parent taxon	Segregation ratio (R:S)${}^{\mathrm{a}}$		$\chi ^2$	$Pr > \chi ^2$
			Observed	Expected		
61S016P006 $\hbox {S}_2$	61S016P006	*F.* $\times$ *ananassa*	77:0	–	–	–
PI612569 $\times$ 12C089P002	PI612569	*F. virginiana* subsp. *grayana*	83:0	–	–	–
12C089P002 $\times$ PI602575	PI602575	*F. chiloensis* subsp. *chiloensis*	35:41	1:1	0.47	0.49
PI552277 $\times$ 12C089P002	PI552277	*F. virginiana* subsp. *virginiana*	54:57	1:1	0.08	0.78
Guardian $\hbox {S}_1$	Guardian	*F.* $\times$ *ananassa*	74:25	3:1	0.00	0.95
Wiltguard $\hbox {S}_1$	Wiltguard	*F.* $\times$ *ananassa*	72:26	3:1	0.12	0.73
Earliglow $\hbox {S}_1$	Earliglow	*F.* $\times$ *ananassa*	80:5	15:1	0.02	0.89
17C327P010 $\hbox {S}_1$	17C327P010	*F.* $\times$ *ananassa*	118:8	15:1	0.00	0.96

${}^\mathrm{a}$The offspring in each segregating population were assigned to resistant (R; $1.0 < y \le 2.0$) and susceptible (S; $2.0 < y \le 5.0$) classes, where *y* was the visual disease symptom rating on a one to five ordinal scale. The resistant parents were hypothesized to either be homozygous (*AA*) or heterozygous (*Aa*) for a partially to completely dominant allele (*A*), whereas the susceptible parents were hypothesized to be homozygous for a recessive allele (*a*). Test statistics were estimated using expected ratios for the segregation of either one dominant resistance gene (1:1 for full-sib and 3:1 for $\hbox {S}_1$ populations) or two unlinked dominant resistance genes with duplicate epistasis (15:1 for $\hbox {S}_1$ populations)

The phenotypes of offspring in each of the segregating populations spanned the entire range from highly resistant ($y = 1$) to highly susceptible ($y = 5$) with bimodal distributions (Online Resource 7 Fig. S4). When individuals within each population were classified as resistant ($1.0 < \bar{y} \le 2.0$) or susceptible ($2.0 < \bar{y} \le 5.0$) using 2.0 as the cutoff on the disease symptom rating scale, the observed phenotypic ratios perfectly fit the expected phenotypic ratios for the segregation of dominant resistance genes (Table 3). The Guardian and Wiltguard $\hbox {S}_1$ and 12C089P002 $\times$ PI602575 and PI552277 $\times$ 12C089P002 full-sib populations each appeared to segregate for a single dominant resistance gene, whereas the Earliglow and 17C327P010 $\hbox {S}_1$ populations appeared to segregate for two dominant genes with duplicate epistasis, where a single dominant allele at either locus was sufficient to confer resistance (Table 3). The statistical inferences were not affected by shifting the cutoff downward to 1.5 or upward to 2.5; hence, we concluded that dominant *R*-genes segregated in these populations (Table 3).

**Fig. 6:**
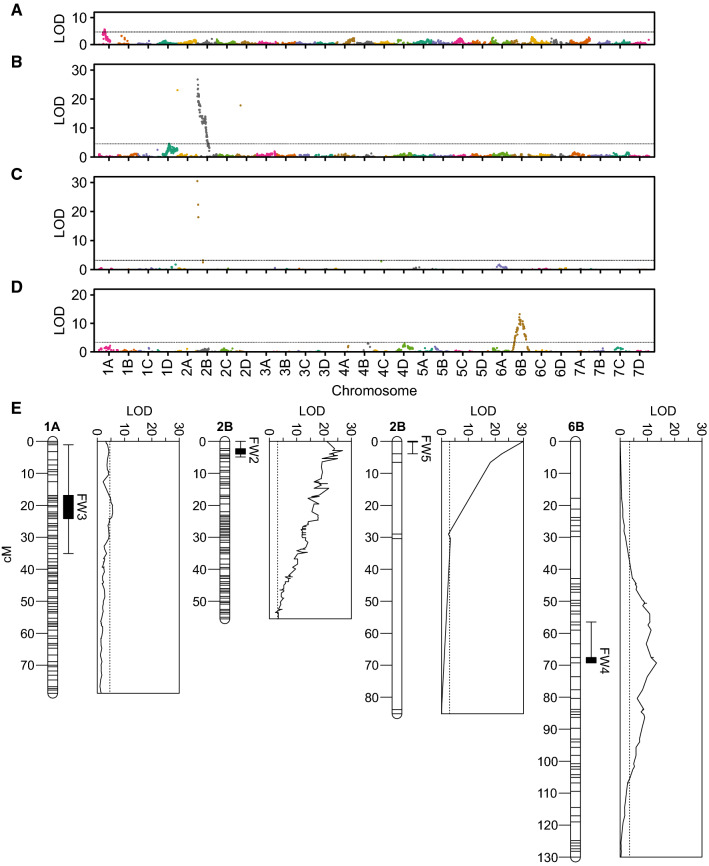
Genome-wide search for associations between SNPs and genes conferring resistance to Fusarium wilt race 1. The upper panels (**A**–**D**) display likelihood odds (LODs) for single marker analyses of associations between SNP marker loci and Fusarium wilt race 1 resistance phenotypes in segregating populations genotyped with the 50K Axiom SNP array. LODs are plotted against physical positions of SNP marker loci in the 'Royal Royce' genome. LODs are shown for the Wiltguard $\hbox {S}_1$ (**A**), Guardian $\hbox {S}_1$ (**B**), 12C089P002 $\times$ PI602575 full-sib (**C**), and PI552277 $\times$ 12C089P002 full-sib (**D**) populations. The lower panel (**E**) displays LODs for analyses of associations between SNP marker loci (plotted along each chromosome) and Fusarium wilt race 1 resistance phenotypes on chromosomes in the Wiltguard $\hbox {S}_1$ (*FW3*), Guardian $\hbox {S}_1$ (*FW2*), 12C089P002 $\times$ PI602575 full-sib (*FW5*), and PI552277 $\times$ 12C089P002 full-sib (*FW4*) populations. The dotted lines specify the $p = 0.05$ significance threshold found by permutation testing ($n = 2,000$). Linkages maps are shown for chromosomes 1A in the Wiltguard $\hbox {S}_1$ population, 2B in the Guardian $\hbox {S}_1$ and 12C089P002 $\times$ PI602575 full-sib populations, and 6B in the PI552277 $\times$ 12C089P002 full-sib population. The box and whisker plots display 1-LOD support intervals (solid box) and 95% Bayes confidence intervals (whiskers) for QTL

### Fusarium wilt resistance loci are found on three non-homoeologous chromosomes

With evidence for the segregation of dominant *R*-genes in the Wiltguard and Guardian $\hbox {S}_1$ and 12C089P002 $\times$ PI602575 and PI552277 $\times$ 12C089P002 full-sib populations, we undertook genome-wide searches for associations between SNP markers and causal loci (Table 3; Online Resource 2). The phenotyped individuals from each population were genotyped with the 50K Axiom SNP array (Hardigan et al. 2020), which yielded genome-wide frameworks of SNP markers anchored to physical positions across the 28 chromosomes (Fig. 6; Online Resources 2 and 7). The average spacing between SNP marker loci ranged from 1.0 to 8.3 cM (Online Resources 2 and 7). The density of SNP marker loci was lower in the parent-specific genetic maps for *F. chiloensis* (PI552277) and *F. virginiana* (PI602575) than for either *F.* $\times$ *ananassa* parent.

Genome-wide searches for SNPs associated with loci segregating for resistance to Fusarium wilt uncovered a single tightly linked cluster of statistically significant SNP markers in each population (Fig. 6A–D). The SNPs most strongly associated with differences in resistance phenotypes were tightly linked to partially to completely dominant *R*-genes that segregated in these populations and mapped to three non-homoeologous chromosomes (Fig. 6; Table 1). The putative *R*-genes are hereafter designated *FW2* (inherited from Guardian), *FW3* (inherited from Wiltguard), *FW4* (inherited from PI552277), and *FW5* (inherited from PI602575) (Fig. 6E).

*FW2* and *FW5* genetically mapped proximal to *FW1* on chromosome 2B (Fig. 6). The effects and positions of the these loci were identified by GWAS (*FW1*) or genetic mapping alone (*FW2* and *FW5*) (Figs. 3, 4 and 6). The SNP markers most strongly associated with each of these loci were different and spanned a 1.6 Mb haploblock (0.21–1.81 Mb) (Figs. 3, 4, 5, 6). To explore the structure of this haploblock in greater depth, we imputed and phased the haplotypes for 71 50K Axiom array-genotyped SNPs among 653 individuals in the California population (Online Resource 6). These included the parents and more distant relatives and common ancestors of the $\hbox {S}_1$ and full-sib progeny that were both genotyped and phenotyped (Fig. 6). Although *FW1*, *FW2*, and *FW5* could be alleles, haplotypes for 71 SNPs within the haploblock predicted to harbor these loci were insufficient to rule out paralogs, and confidence intervals for the estimated positions of these loci spanned the gene-rich haploblock (Online Resource 6). The SNP haplotypes for Fronteras and Portola were identical except for three consecutive SNPs in a short haploblock (670,184–698,410 bp) slightly downstream of the location predicted to harbor *FW1* (488,699–636,061 bp). The haplotype associated with the dominant *FW1* allele for that haploblock was ascertained from the genotypes of Fronteras and Portola (Online Resource 6). The haplotypes observed for the other resistant parents (e.g., donors of *FW2* and *FW5*) differed from each other and Fronteras and Portola; hence, from SNP haplotypes and approximate physical positions, we could not unequivocally show that the putative *R*-genes associated with these phenotypically mapped loci were allelic. Finally, the KASP assays we developed for informative SNPs associated with these loci were not cross-predictive (Table 2). The paralog hypothesis seems plausible for the underlying *R*-genes; however, our data were insufficient to rule out the single locus, multiple allele hypothesis. Although additional studies are needed to resolve this question, the classes of *R*-genes hypothesized to underlie these loci (see below) are commonly found in tandemly duplicated clusters in plants (Leister et al. 1998; Cook et al. 2012; Muñoz-Amatriaín et al. 2013; Alcázar et al. 2014; Dolatabadian et al. 2017; Lye and Purugganan 2019; Van de Weyer et al. 2019).

The genotypic means, effects, and PVE estimates for SNP markers tightly linked with *FW2* and *FW5* were nearly identical to estimates for SNP markers associated with *FW1* in the Fronteras and Portola $\hbox {S}_1$ populations (Table 1). *FW2* was nearly completely dominant ($d/a = 0.85$). The additive and dominance effects of the *FW2* locus were 1.5- to 1.9-fold greater than those reported for the *FW1* locus, partly because unfavorable (susceptible) allele homozygotes were more strongly susceptible in the Guardian $\hbox {S}_1$ population than in the Fronteras and Portola $\hbox {S}_1$ populations. The estimated marginal means (EMMs) for favorable (resistant) allele homozygotes ranged from 1.07 to 1.25 in the three populations (Table 1). The EMM for resistant homozygotes (*FW2FW2* = *AA*) was $\bar{y}_{\textit{AA}} = 1.1$, whereas the EMM for susceptible homozygotes (*fw2fw2* = *aa*) was $\bar{y}_{\textit{aa}} = 4.5$. We could not estimate the degree of dominance for *FW5* because the *AA* homozygote was not observed in the full-sib population; however, the EMM for the heterozygote was 1.06, which implies that the *FW5* allele might be completely dominant.

Although the statistical evidence for the segregation of a single dominant *R*-gene on chromosome 1A was strong in the Wiltguard $\hbox {S}_1$ population, the effects of SNPs associated with *FW3* were weaker than those associated with *FW1*, *FW2*, and *FW5* on chromosome 2B (Table 1). The most significant *FW3*-associated SNP was AX-123363542 (LOD = 5.6), which only explained 23% of the phenotypic variation for resistance to race 1. Despite this, the EMM for *FW3* homozygotes ($\bar{y}_{\textit{AA}} = 1.53$) was only slightly greater than the EMMs for *FW1* and *FW2* homozygotes ($\bar{y}_{\textit{AA}} = 1.1$ and $\bar{y}_{\textit{AA}} = 1.2$, respectively). Although the PVE estimate was greater for *FW4* (42.9%) than *FW3* (22.6%), the EMMs for SNP marker heterozygotes were virtually identical: 1.76 for *FW3* and 1.69 for *FW4*; hence, *FW4* appears to be as strong as *FW3* (Table 1).

### Pathogen defense genes associated with Fusarium wilt resistance loci

With the genomic locations of *FW1*, *FW2*, and *FW5* narrowed to a short haploblock on chromosome 2B (Fig. 4), we searched annotations in the 'Royal Royce' reference genome (FaRR1; Hardigan et al. 2021b) to identify genes encoding proteins known to play an important role in race-specific disease resistance via pathogen recognition and activation of defense responses, e.g., pathogen-associated molecular pattern (PAMP)-triggered immunity or effector triggered immunity (ETI) (Hammond-Kosack and Jones 1996, 1997; Jones et al. 2016; Zhang and Coaker 2017; Lolle et al. 2020). Eight of 1,208 annotated genes found in the 0.0-5.0 Mb haploblock on chromosome 2B encode proteins with known *R*-gene domains and functions (Table 4). Three of the eight were found in the haploblock predicted to harbor *FW1*. These included one coiled-coil domain (CC) NLR encoding gene (517,947-521,932 bp) and two tightly linked Toll-interleukin 1 receptor domain (TIR) type NLR encoding genes (1,176,817-1,197,734 bp) (Table 4). Hence, the most promising candidate genes for *FW1* encode NLR proteins.

**Table 4:** Pathogen defense genes in linkage disequilibrium with Fusarium wilt resistance loci

Locus	Annotation${}^{\mathrm{a}}$	Chr${}^{\mathrm{b}}$	Position${}^{\mathrm{c}}$		AT_Gene${}^{\mathrm{d}}$	Domain${}^{\mathrm{e}}$	Protein Family${}^{\mathrm{f}}$
			Start	Stop			
*FW1*	Fxa2Bg200055	2B	517,947	521,932	AT3G07040.1	NB-ARC	Disease resistance protein (CC-NBS-LRR)
*FW1*	Fxa2Bg200141	2B	1,176,817	1,177,501	AT5G36930.1	NB-ARC	Disease resistance protein (TIR-NBS-LRR)
*FW1*	Fxa2Bg200143	2B	1,191,345	1,197,734	AT4G12010.1	NB-ARC	Disease resistance protein (TIR-NBS-LRR)
*FW1*	Fxa2Bg200175	2B	1,417,268	1,420,357	AT1G09970.1	LRR	Receptor-like protein
*FW1*	Fxa2Bg200271	2B	2,147,842	2,150,028	AT3G43740.1	LRR	Receptor-like protein
*FW1*	Fxa2Bg200289	2B	2,230,151	2,231,286	AT3G07040.1	NB-ARC	Disease resistance protein (CC-NBS-LRR)
*FW1*	Fxa2Bg200404	2B	3,254,299	3,257,893	AT4G34220.1	LRR	Receptor-like kinase
*FW1*	Fxa2Bg200412	2B	3,333,973	3,335,142	AT2G15320.1	LRR	LRR family protein
*FW3*	Fxa1Ag101064	1A	6,037,746	6,048,242	AT5G17680.2	NB-ARC	Disease resistance protein (TIR-NBS-LRR)
*FW3*	Fxa1Ag101128	1A	6,419,064	6,423,914	AT5G66330.1	LRR	Receptor-like kinase
*FW3*	Fxa1Ag101177	1A	6,682,945	6,685,470	AT3G50950.1	NB-ARC	Disease resistance protein (CC-NBS-LRR)
*FW3*	Fxa1Ag101301	1A	7,378,435	7,381,245	AT3G59410.1	Kinase	Protein kinase
*FW3*	Fxa1Ag101393	1A	7,976,850	8,032,406	AT5G66900.1	NB-ARC	Disease resistance protein (CC-NBS-LRR)
*FW3*	Fxa1Ag101404	1A	8,089,827	8,100,427	AT5G66900.1	NB-ARC	Disease resistance protein (CC-NBS-LRR)
*FW4*	Fxa6Bg102048	6B	15,186,176	15,189,317	AT3G57830.1	LRR	Receptor-like kinase
*FW4*	Fxa6Bg102076	6B	15,542,705	15,545,819	AT5G06940.1	LRR	Receptor-like kinase
*FW4*	Fxa6Bg102106	6B	15,792,021	15,794,795	AT2G41820.1	LRR	Receptor-like kinase

${}^{\mathrm{a}}$Annotated gene name in the 'Royal Royce' genome (Hardigan et al. 2021b)

${}^{\mathrm{b}}$Chromosome (Chr) numbers follow the nomenclature proposed by Hardigan et al. (2021a)

${}^{\mathrm{c}}$Physical position of the annotated gene in the 'Royal Royce' genome

${}^{\mathrm{d}}$The Arabidopsis Information Resource gene identification number (https://www.arabidopsis.org/index.jsp)

${}^{\mathrm{e}}$Domain architecture abbreviations are nucleotide binding-ARC (NB-ARC) and leucine rich repeat (LRR)

${}^{\mathrm{f}}$The protein family abbreviations are coiled-coil nucleotide binding site-LRR (CC-NBS-LRR) and Toll-interleukin 1 receptor NBS-LRR (TIR-NBS-LRR)

The approximate 95% Bayes confidence interval for the genomic location of *FW4* on chromosome 6B (13.8–16.3 Mb) was fairly wide and consequently harbored 197 annotated genes in the 'Royal Royce' reference genome (Table 4). Nine of these 197 annotated genes are predicted to encode *R*-proteins that mediate gene-for-gene resistance in plants (Hammond-Kosack and Jones 1996; Jones et al. 2016; Zhang and Coaker 2017; Lolle et al. 2020). These included multiple NBS-LRR *R*-proteins (Table 4). Finally, the approximate 95% Bayes confidence interval for the genomic location of *FW3* on chromosome 1A (4.8 to 8.1 Mb) was slightly wider than that observed for the other mapped loci because the effect of the locus was weaker. There were 535 annotated genes within that interval, of which seven were predicted to encode NLR or other *R*-proteins (Table 4). This was the locus with the weakest support for the segregation of a race-specific *R*-gene; however, as noted earlier, homozygous resistant (*FW3FW3*) offspring in the Wiltguard $\hbox {S}_1$ population were highly resistant (EMM = 1.53). Hence, even if *FW3* does not encode a race-specific *R*-protein, this locus merits further study, in part because the favorable allele (*FW3*) can be deployed and pyramided to increase the durability of resistance to Fusarium wilt.

### High-throughput SNP genotyping assays for marker-assisted selection of Fusarium wilt resistance loci

To accelerate the introduction and selection of Fusarium wilt resistance genes in breeding programs, we developed a collection of high-throughput Kompetitive Allele Specific PCR (KASP) markers for SNPs in linkage disequilibrium with *FW1*-*FW5* (Table 2; Fig. 5). Collectively, 25 KASP markers were designed for the five loci using PolyOligo 1.0 (https://github.com/MirkoLedda/polyoligo). The genotypic clusters for 17 of these were codominant (non-overlapping), co-segregated with the predicted resistance loci, and were robust and reliable when tested on diverse germplasm accessions (Fig. 5; Online Resource 3). For each target locus, at least one KASP-SNP marker had a prediction accuracy in the 98-100% range when tested in the original populations where they were discovered (Table 2; Fig. 5). To further gauge their accuracy when applied in diverse germplasm, they were genotyped on 78 California and 66 non-California individuals, mostly cultivars (Online Resources 1 and 3). Because the causal genes and mutations underlying *FW1*-*FW5* are not known, the SNPs we targeted are highly population-specific (Table 2). They are strongly predictive when applied in populations where specific genes are known to be segregating and moderately predictive when assayed among random samples of individuals because of recombination (LD decay) between the SNP markers and unknown causal mutations.

## Discussion

The deployment of Fusarium wilt resistant cultivars has become critical in California since the early 2000s when outbreaks of the disease were first reported (Koike et al. 2009; Koike and Gordon 2015). This disease has rapidly spread and become one of the most common biotic causes of plant death and yield losses in California, the source of 88-91% of the strawberries produced in the US (http://www.agmrc.org/commodities-products/fruits/strawberries; https://www.nass.usda.gov/). The scope of the problem was initially unclear, as were the solutions, because the resistance phenotypes of commercially important cultivars, genetic mechanisms underlying resistance, and distribution and race structure of the pathogen were either unknown or uncertain when the disease unexpectedly surfaced in California (Koike and Gordon 2015; Pincot et al. 2018). A breeding solution instantly emerged with the discovery of *FW1* (Pincot et al. 2018), and was further strengthened with the discovery of additional homologous and non-homoeologous resistance genes in the present study (Fig. 6; Table 1). Genetic and physical mapping of these race-specific *R*-genes has enabled the rapid development and deployment of Fusarium wilt resistant cultivars through marker-assisted selection. The transfer of *R*-genes from race 1 resistant donors to susceptible recipients via MAS has been rapid because the resistant alleles are dominant, found in both heirloom and modern cultivars, and identifiable without phenotyping using SNP markers tightly linked to the causal loci (Online Resources 1 and 3; Table 2; Fig. 4).

Once *FW1* was discovered, we knew that we had a robust solution to the race 1 resistance problem; however, we had virtually no knowledge of the diversity of Fusarium wilt *R*-genes in populations of the wild octoploid progenitors and heirloom cultivars of cultivated strawberry that might be needed to cope with pathogen race evolution (Pincot et al. 2018; Henry et al. 2021). We did not purposefully set out to identify redundant *R*-genes but rather to scour global diversity for ancestrally diverse *R*-genes, both to facilitate *R*-gene pyramiding (Poland and Rutkoski 2016; van Wersch et al. 2020; Chitwood-Brown et al. 2021) and inform future searches for sources of resistance to as yet unknown races of the pathogen, in addition to assessing the frequency, diversity, and distribution of *R*-genes in the wild and domesticated reservoirs of genetic diversity (Fig. 1; Online Resource 1). Our results paint a promising picture for the identification of genes for resistance to race 2 and other as yet unknown races of the pathogen. As our phenotypic screening studies showed, the frequency of resistance to race 2 was comparable to that observed for race 1 (Online Resource 1; Henry et al. 2021). Similar to our findings for race 1, the sources we identified for resistance to race 2 were symptomless, which suggests that gene-for-gene resistance might underlie their phenotypes. The genetic basis of resistance to race 2 and other races of the pathogen, however, has not yet been elucidated. There is empirical evidence that resistance to Australian isolates of the pathogen might be quantitative (Mori et al. 2005; Paynter et al. 2014). Henry et al. (2021) showed that the non-chlorotic symptom syndrome caused by Australian Fof isolates (wilt-*fragariae*) differs from the chlorotic symptom syndrome caused by California and Japanese Fof isolates (yellows-*fragariae*). Hence, the genetic basis of resistance to the wilt- and yellows-*fragariae* diseases could be markedly different. We identified several strong sources of resistance to Australian and other non-California isolates of the pathogen that should accelerate the discovery of novel race-specific *R*-genes, elucidation of genetic mechanisms, and development of resistant cultivars (Online Resource 1; Henry et al. 2021).

Growing resistant cultivars is indisputably a highly effective and cost-free method for preventing losses to Fusarium wilt race 1 in strawberry (Table 1; Fig. 6). We estimate that approximately two-thirds of the cultivars grown in California since the earliest outbreaks in 2005 were highly susceptible, whereas the other one-third were highly resistant (Pincot et al. 2018; Online Resource 1). Using the race 1 resistance phenotypes observed in our studies and California Strawberry Commission production statistics (https://www.calstrawberry.com/en-us/market-data/acreage-survey), we discovered that susceptible cultivars have been planted on 49-85% of the acreage in California over the last eleven years (2010-2021). That percentage has hovered between 55 and 59% since 2014. Hence, susceptible cultivars continue to be widely planted in California despite incontrovertible evidence that losses to the disease can be prevented by planting cultivars carrying one of the race-specific *R*-genes we identified (Table 1; Fig. 6; Koike and Gordon 2015; Pincot et al. 2018). Over five years of screening plants that were either naturally infected or artificially inoculated with race 1 isolates of the pathogen, we have not observed visible symptoms on cultivars or other germplasm accessions carrying the dominant *FW1* allele (Online Resource 1; Pincot et al. 2018; Henry et al. 2021). Although private sector cultivars were unavailable for inclusion in our studies, the prevalence of *R*-genes in publicly available germplasm collections and shared ancestry of public and private sector cultivars worldwide suggests that the same *R*-genes are widely found in private sector cultivars (Pincot et al. 2021; Hardigan et al. 2021a). We anticipate that production in California will ultimately shift away from susceptible cultivars, particularly as the incidence of the disease increases and yield losses mount (Koike and Gordon 2015; Henry et al. 2019).

The cloning and characterization of *R*-genes underlying race-specific resistance is important for developing an understanding of their function and interactions with the pathogen and building the foundation needed to engineer resistance through genome editing or other approaches (Chisholm et al. 2006; Chiang and Coaker 2015; Dong and Ronald 2019; Lolle et al. 2020; van Wersch et al. 2020). The *I* genes that confer race-specific resistance to *F. oxysporum* f. sp. *lycopersici* in tomato differ in durability and function and provide a model for future studies in strawberry (Bohn and Tucker 1939; Sela-Buurlage et al. 2001; Houterman et al. 2009; Catanzariti et al. 2015, 2017). The *I-2* gene had a significantly longer life span than the *I* gene, which was defeated in less than a decade subsequent to deployment (Bohn and Tucker 1939; Alexander 1945). The durability differences of the tomato *I* genes have been attributed to differences in the dispensability of avirulence genes or mutations in avirulence genes that defeat known *R*-genes (Catanzariti et al. 2015, 2017). The life spans of the race-specific *R*-genes we identified in strawberry are of course unknown; however, the sheer abundance and diversity of race 1 *R*-genes found in the wild relatives predict that sources of resistance to other races of the pathogen can be rapidly identified and deployed (Fig. 1; Table 1; Online Resource 1). The earliest reports of resistance to California isolates of the pathogen emerged when the disease initially surfaced in field experiments in California where well known cultivars were being grown (Koike et al. 2009; Koike and Gordon 2015). We have since shown that the resistance phenotypes of those cultivars were mediated by *FW1*; hence, the *FW1* gene has endured for at least 16 years to-date. Our search for diverse *R*-genes was partly motivated by the need to prepare for the havoc created by the inevitable evolution and emergence of novel pathogen races and inadvertent introduction of foreign races of the pathogen through infected plants or soil (Gordon 2017; Henry et al. 2017, 2021).

Our findings suggest that the resistant germplasm accessions identified in the present study carry one or more dominant Fusarium wilt *R*-genes, and that race 1 *R*-genes are found in a wide range of heirloom and modern cultivars (Tables 1–3; Figs. 1, 2; Online Resource 1). The latter finding further suggests that race 1 resistance genes are found in domesticated populations worldwide, albeit often at low frequency because they have not been consciously selected, e.g., we previously showed that the frequency of *FW1* was 0.16 in the pre-2015 California population and that *FW1* originated in Shasta and other cultivars released in the 1930s (Pincot et al. 2018), 70 to 80 years before Fusarium wilt was first reported in California (Koike et al. 2009; Koike and Gordon 2015). The phenotypic and pedigree databases we developed should expedite the identification and incorporation of Fusarium wilt resistance into modern cultivars (Online Resource 1; Fig. 2; Pincot et al. 2021). Our data suggest that a certain percentage of modern cultivars are bound to fortuitously carry Fusarium wilt *R*-genes. That was exactly what we discovered in the California population (Pincot et al. 2018).

The race 1 *R*-genes we identified in cultivated strawberry are predicted to be a small sample of those found in the wild reservoir of genetic diversity (Fig. 1; Tables 1, 2, 3; Online Resource 1). Our analyses of the pedigree records of heirloom and modern cultivars show that those *R*-genes were fortuitously introduced through early founders and survived breeding bottlenecks predating the late twentieth century emergence of this disease in strawberry (Fig. 2; Winks and Williams 1965; Koike et al. 2009; Pincot et al. 2021). Their chance survival in individuals that dominate the ancestry of domesticated populations worldwide is noteworthy because artificial selection for resistance to Fusarium wilt was not consciously applied anywhere outside of Australia or Japan until 2015 when we initiated breeding for resistance to California-specific isolates of the pathogen (Mori et al. 2005; Paynter et al. 2014, 2016; Pincot et al. 2018). This suggests that genes conferring resistance to Fusarium wilt were fairly common in the founders, which is what our data showed—52% of the wild octoploid individuals screened in the present study were highly resistant to race 1 and predicted to carry dominant race 1 *R*-genes (Online Resource 1). Our analyses of pedigree records further suggest that many of the race 1 *R*-genes found in heirloom and modern cultivars have flowed through common ancestors and thus could be identical-by-descent (Fig. 2; Pincot et al. 2021).

We cast a wide net in our original phenotypic screening experiments because the genetic basis of resistance was unknown before race-specific *R*-genes were discovered (*FW1*-*FW5*), knowledge was lacking to strategically narrow the search, and the frequency of resistance among accessions preserved in public germplasm collections was unknown. Our phenotypic screens were designed by assuming that we might be searching for a needle in a haystack, primarily because phenotypic screens in tomato and other plant species had shown that genes conferring resistance to Fusarium wilt were uncommon or only found in wild relatives, e.g., the Fusarium wilt *R*-genes in cultivated tomato were transferred from wild relatives (Bohn and Tucker 1939; Alexander 1945; Sela-Buurlage et al. 2001; Catanzariti et al. 2015, 2017). Although the domestication and breeding histories of tomato and strawberry are quite different, the frequencies of Fusarium wilt resistance among accessions of wild relatives are similar. Fifty-two to 57% of the individuals we sampled from wild populations of *F. chiloensis* and *F. virginiana* were resistant to races 1 and 2, which are comparable to the percentages reported for race-specific *R*-genes in the wild relatives of tomato (Bohn and Tucker 1939; Bournival et al. 1990; Bournival and Vallejos 1991; Sela-Buurlage et al. 2001).

Wild relatives could certainly become an important source of *R*-genes in strawberry breeding going forward; however, the prevalence of *R*-genes in modern cultivars circumvents the need to introduce alleles from wild relatives or exotic sources, which has often been necessary for the development of Fusarium wilt resistant cultivars in tomato, cotton, and other plants (Sela-Buurlage et al. 2001; Ulloa et al. 2013). The wild relatives of many of the agriculturally important species impacted by this pathogen carry chromosome rearrangements or structural DNA variation that impedes gene flow and the recovery of recombinants, e.g., the tomato *I* genes have been introgressed from wild relatives with interspecific structural variation that suppresses recombination and causes the persistence of unfavorable alleles through linkage drag (Scott and Jones 1989; Sela-Buurlage et al. 2001; Hemming et al. 2004; Takken and Rep 2010). Notably in strawberry, the octoploid progenitors are inter-fertile and have highly syntenic genomes with no known or apparent barriers to gene flow or suppressed recombination in wide crosses (Darrow 1966; Hardigan et al. 2020). This does not eliminate the linkage drag problem altogether but simplifies the challenge of purging unfavorable alleles introduced by exotic donors in wide crosses (Young and Tanksley 1989; Fulton et al. 2000). Moreover, cultivated strawberry has emerged from only 250 years of domestication in interspecific hybrid populations between wild relatives and thus has not experienced population bottlenecks on a scale similar to wheat, tomato, and other staples that have undergone 7000 to 10,000 years of domestication (Darrow 1966; Hardigan et al. 2020; Pincot et al. 2021).

## Data Availability

The data for these studies are publicly available in the online resources and a Dryad repository (https://doi.org/10.25338/B86057). Custom scripts developed for genetic mapping were deposited in the Dryad repository. Online Resource 1 is a database with Fusarium wilt resistance phenotypes (estimated marginal means) and passport data for individuals screened for resistance to different isolates of *Fusarium oxysporum* f. sp. *fragariae*. Online Resource 2 is a database with QTL mapping statistics and genetic positions (cM) and linkage groups for *de novo* genetically mapped 50K Axiom SNP marker loci in the Guardian $\hbox {S}_1$, Wiltguard $\hbox {S}_1$, PI552277 $\times$ 12C089P002, and 12C089P002 $\times$ PI602575 mapping populations. Online Resource 3 is a database with physical positions, primer sequences, and other data for KASP-SNP markers. Online Resource 4 is a database for individuals in the UCD population with genotypes for 50K Axiom arrays SNPs in the haploblock on chromosome 2B predicted to harbor *FW1*. Online Resource 5 is a pedigree database for 141 individuals in the non-UCD population and the UCD cultivar ‘Wiltguard’. Online Resource 6 is a database with haplotypes for 71 50K Axiom array-genotyped SNPs in LD with Fusarium wilt resistance loci (*FW1*, *FW2*, and *FW5*) on chromosome 2B. Online Resource 7 contains supplemental figures and tables, as well as a table of contents for Online Resources. Online Resource 8 is a database with GWAS statistics estimated using the physical positions of 50K or 850K Axiom SNP markers in the ‘Camarosa’ and ‘Royal Royce’ reference genomes.
